# The “Netweave-Approach”—A Platform Combining Sociology, Resource Management and Psychology for Networking Conservation Stakeholders

**DOI:** 10.1007/s00267-025-02268-1

**Published:** 2025-08-30

**Authors:** Felix Przesdzink, Marvin Frede, Florian Fiebelkorn

**Affiliations:** 1 Department Didactics of Biology, Osnabrück University, Osnabrück, Germany; 2 Department Didactics of Biology, Osnabrück University, Münster, Germany

**Keywords:** Environmental networking, Stakeholder collaboration, Social network analysis, Conservation management, Organizational culture, Resource-based networking

## Abstract

The “Netweave Approach” innovatively combines constructs from sociology, resource management, organizational psychology, and environmental psychology within a single framework and corresponding management tool to support regional management in facilitating effective interactions among environmental stakeholders. This interdisciplinary approach, uniting social network analysis, resource mapping, organizational culture assessment, environmental worldview evaluation and environmental risk perception measurement in a way previously unused, enables the development of a uniquely detailed database of environmental stakeholders. Through this system, regional managers can gain detailed insights into their stakeholder networks, which provides a robust foundation for a network consultation process that maximizes effective regional collaboration. In this paper, we present the theoretical foundations of the approach and the structure of the resulting framework and management tool. Furthermore, we explain the concept of network consultation operating in conjunction with the tool. As a practical summary, we compile the results of a preliminary implementation of a corresponding stakeholder platform in the city and district of Osnabrück in northwestern Germany. Finally, we discuss whether the Netweave Approach can be adapted to various regions and contexts, contributing to more coordinated and effective conservation initiatives in diverse regional contexts. We provide specific suggestions regarding aspects of the approach that would need to be adjusted when applied to other regions.

## Introduction

We are currently experiencing the sixth major mass extinction event in Earth’s history, indicated by the transgression of six out of nine planetary boundaries (Díaz et al. 2019; Richardson et al. 2023). Global targets, such as the Sustainable Development Goals, aim to address this anthropogenic crisis, yet effective, on-the-ground implementation requires the agreement and coordination of a diverse array of stakeholders, including resource users, conservationists, public authorities, and research groups (Jasny et al. 2021). While external factors like funding and governmental structures impact regional conservation and sustainable development (Bazzoli et al. 2003; Vance-Borland and Holley 2011), productive stakeholder interactions and participation in planning and decision-making are often crucial to success (Bodin and Crona 2009; Guerrero et al. 2013; Knight et al. 2006; Schuett et al. 2001; Vance-Borland and Holley 2011). Transdisciplinary collaborations offer unique opportunities to address complex socio-ecological issues by integrating traditional, practical, and scientific knowledge (Balmford and Cowling 2006; Bazzoli et al. 2003; Fischer et al. 2015; Knight et al. 2006). Conversely, excessive homogeneity within groups can limit access to new resources and knowledge (Krackhardt and Stern 1988), while land-use conflicts and incompatible stakeholder views may obstruct sustainable environmental management (Bazzoli et al. 2003; Luyet et al. 2012; Reed et al. 2009).

The need for intensive, low-conflict networking among environmental stakeholders is often constrained by limited time and human resources available for collaboration and networking—whether in voluntary work, public authorities, or research institutions (Blackman 2009; Geng et al. 2022; Latter et al. 2024; Luyet et al. 2012; Przesdzink et al. 2021; Zubair 2001). Networking concepts for regional environmental stakeholders must therefore be as time-efficient as possible to enable participation despite high workloads. When hosted by a public authority—often a practical choice given their central role in regional stakeholder networks (Ernstson et al. 2009; Herzog 2020; Nita et al. 2018; Przesdzink et al. 2021)—the approach should also require minimal time investment from these users. In regions with numerous stakeholders (e.g., over 190 organizations in our pilot region), a systematic and standardized approach is essential for efficient coordination.

In the “Netweave” project, on which this paper is based, we developed the “Netweave Approach” to meet these criteria by integrating methods from sociology, resource management, and psychology:

1. A social network analysis maps current interactions among stakeholders across the dimensions of “acquaintance,” “collaboration,” and “conflict.”
2. A stakeholder analysis focusing on existing and required resources identifies mutually beneficial potential collaborations.
3. An analysis of stakeholders’ organizational cultures assesses the compatibility of their working methods and practices.
4. Finally, analyzing environmental worldviews and risk perceptions evaluates the ideological alignment of stakeholders regarding environmental issues.

In addition to these analytical components, we also collect basic information about each stakeholder, including contact details, the spatial and thematic scope of their activities, criteria for or against potential collaborations, and—where relevant—typical allies and adversaries. These data are obtained through stakeholder interviews or standardized online questionnaires and stored in a MariaDB database, either manually (for interviews) or automatically (for questionnaires). The database, accessible via a Laravel-based web application, enables a centrally established network consultation team to systematically connect organizations based on the compatibility of the information provided.

The Netweave Approach provides a systematic and replicable procedure for network managers to identify suitable collaboration partners based on empirical data. The digital platform functions as a centralized data infrastructure that facilitates onboarding of new staff and ensures continuity during generational transitions. For stakeholder organizations, the approach reduces the effort typically associated with establishing new contacts by offering a structured and data-driven process for identifying potentially compatible partners. In the following sections, we discuss the theoretical foundations, including Social Network Analysis (SNA), Organizational Culture Assessment, and Environmental Worldview Analysis, to provide the conceptual framework for the study. This is followed by an overview of the pilot region, the city and district of Osnabrück, to contextualize the application of the Netweave Approach. The data collection methodologies as well as structure and functionality of the resulting stakeholder platform and database are then detailed, alongside the processes involved in networking consultancy. Finally, the paper evaluates the pilot implementation of the Netweave Approach, highlighting its successes, challenges, and potential for transferability to other regions, with suggestions for future development and application.

## Theoretical Background

### Social Network Analysis

SNA is a sociological approach that examines relationships and structures within networks, focusing on how nodes (e.g., individuals, organizations, or species) interact to understand the structure and dynamics of these interactions (Wasserman and Faust 1994). In conservation and resource management, SNA is used to map and analyze complex stakeholder relationships. By highlighting key actors and their connections, SNA helps identify influential entities that can drive regional conservation efforts (Cohen et al. 2012; Ernstson et al. 2010). Structural analyses of social networks allow for interpreting properties within stakeholder networks, such as network density or mutually separated subgroups, in the context of effective regional conservation (Bodin et al. 2017; Friedman et al. 2020; Mbaru and Barnes 2017; Morgans et al. 2017).

The Netweave Approach uses SNA to map the current relationships among regional stakeholders across the dimensions of “acquaintance” (both stakeholders know each other), “collaboration” (both have worked together on a project in the last 3 years; includes “acquaintance”), and “conflict” (both were involved in a conflict in the last 3 years; includes “acquaintance” and can overlap with “collaboration”). These data are valuable for individual stakeholder networking: If contact or even a previous collaboration already exists, a brief information to both stakeholders is sufficient to enable collaboration. If the two stakeholders do not know each other or are in conflict with each other, more complex interventions are necessary to initiate collaboration.

**Fig. 1:**
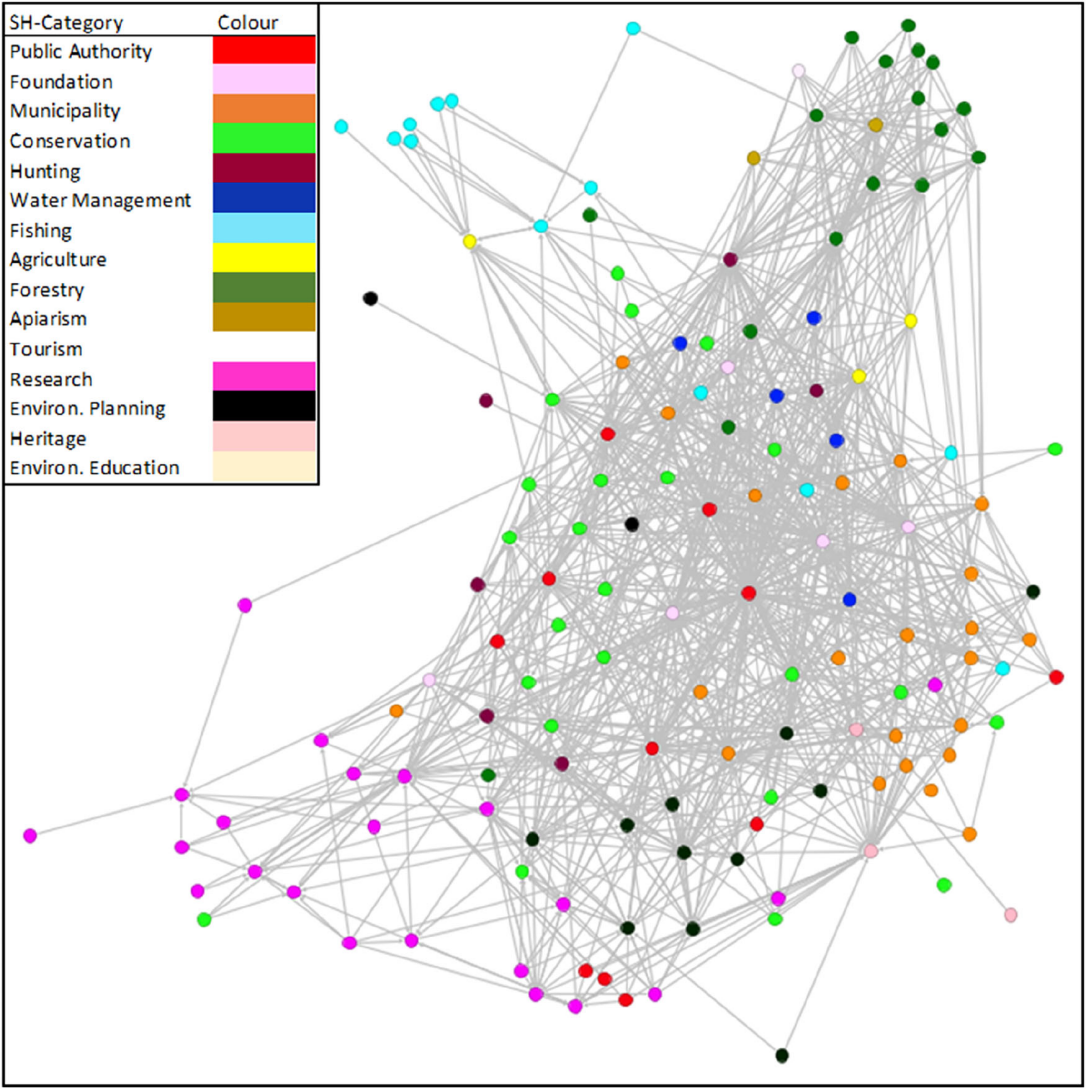
Example of a Social Network Graph. Graphic representation of the “collaboration” ties between stakeholders in our pilot region who completed our online questionnaire at the time of compilation in September 2024. The graphic was created with Gephi 0.10.1. Top left is the legend for color coding by different stakeholder categories. Very peripheral to the network, accumulations of actors from the fields of research (bottom left, pink), fisheries (top left, light blue) and forestry (top right, dark green) can already be seen with the naked eye. Such a graphic can be used to illustrate to individual players their own position in the network or to give regional management an overview of structural phenomena in the network. Further statistical analyses would be required for any quantitative statements

Additionally, using programs such as Gephi (Bastian et al. 2009), network data can be extracted from the Netweave Platform and visualized in a network graph, providing a “map” of the network that stakeholders can use to see their position within the regional network, while regional managers gain a graphic overview of current stakeholder interactions (Fig. 1). Observing a network map can reveal phenomena such as central actors with numerous connections or subgroups that are separated from the main network. Further analysis, if appropriate, can yield quantitative statistical data on actors with various functional centralities (Cohen et al. 2012; Ernstson et al. 2010; Przesdzink et al. 2021). For instance, Degree Centrality identifies actors with the most direct connections, indicating which stakeholders serve as key points of contact within the network. Betweenness Centrality, on the other hand, highlights actors positioned on the shortest paths between others, marking them as essential bridges for connecting different groups and facilitating information flow. Additionally, network analysis can highlight communities of actors poorly connected to the rest of the network (Girvan and Newman 2002; Newman and Girvan 2004; Przesdzink et al. 2021).

However, a sole focus on the structural aspects of a stakeholder network overlooks the qualitative significance of connections within it. Given the limited time resources of all stakeholders, it is essential to consider whether connecting every isolated group to the broader network and creating networks as dense as possible truly adds value. Due to the transaction costs associated with maintaining contacts (Boschet and Rambonilaza 2018; Gallemore et al. 2015), a maximally dense network may not necessarily be a “good” network from the stakeholders’ perspective. To identify which (potential) interactions genuinely add value for stakeholders, it is necessary to assess their available and required resources, which can be achieved through Stakeholder Analysis (SA).

### Stakeholder Analysis—Resources

SA is a holistic approach for gathering information about stakeholders to understand their behavior, intentions and relations as well as to assess their influences and resources (Reed et al. 2009; Varvasovszky and Brugha 2000). The approach originates from political economy and management science (Grimble and Wellard 1997) and ensures that groups and interests of all stakeholders are fairly represented (Prell et al. 2009). It is particularly useful in complex situations in which the compatibility of interests of the involved is hampered and, thus, is frequently applied in the field of natural resource management (Grimble and Wellard 1997; Vogler et al. 2017). In this context, SA has been used primarily as an instrument for achieving specific goals, such as including stakeholders in a particular decision-making process (Reed et al. 2009) or as a decision-support tool (Bendtsen et al. 2021). Overall, the concept of SA is very flexible and can be adapted to the respective research or project context. It can, but does not necessarily have to, include all of the aspects mentioned above.

The Netweave Approach collects data on the availability of and the requirement for resources of the individual stakeholders. In our case, “resources” include, among other things, knowledge (typically specialized biological expertise in various categories, such as peatlands or birds), bureaucratic skills, financial resources, equipment, manpower, or project areas. A complete list can be found in the Supplementary Material “List of Resource Categories”. These resource categories reflect our pilot platform’s focus on conservation stakeholders in our pilot region. In other regions or thematic segments (e.g., climate protection), different categories would need to be selected independently (see Transferability). Using this data, it is possible to compare whether two stakeholders in the network possess a resource that each other requires—in this case, collaboration would be mutually beneficial. Data from the SNA can be used complementary to compare the current relationship between the two stakeholders and establish contact accordingly.

However, the members of stakeholder organizations are human beings whose actions are influenced by various factors, including psychological aspects such as the organizational culture they work in or their environmental worldview. Numerous studies emphasize the importance of focusing on these “human” aspects (Bennett et al. 2017; Niemiec et al. 2021; Sanborn and Jung 2021), including trust (Young et al. 2016), values (Biggs et al. 2011; Crow and Baysha 2013), and attitudes (Ansong and Røskaft 2011; Ray and Bhattacharya 2013). The influence of such psychological factors on interactions among stakeholder representatives was noted in our data acquisition interviews and a previous study in our pilot region (Przesdzink et al. 2024–1), using colloquial terms such as “harmonizing” or “being on the same wavelength.” The Netweave Approach employs organizational and environmental psychology frameworks to replace these colloquial expressions with specific scientific constructs.

### Stakeholder Analysis—Organizational Cultures

**Fig. 2:**
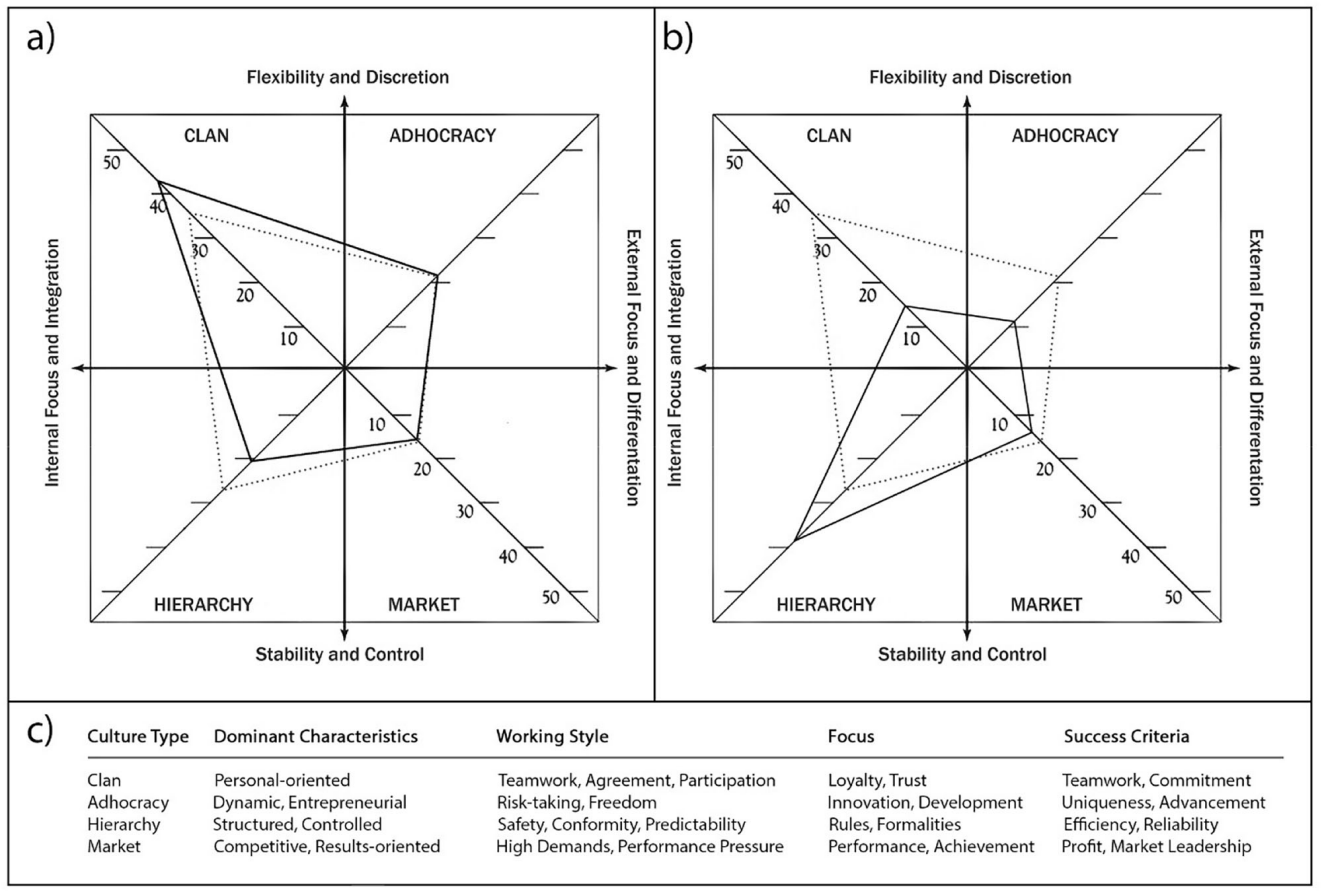
Representation of the concept of organizational culture. **a** Organizational culture profile of a hunting association from our pilot platform. The black line represents the profile of the association, while the red dashed line indicates the average values of all profiles recorded in the platform. The dominant Clan culture is evident, emphasizing Internal Focus and Integration as well as Flexibility and Discretion. **b** Organizational culture profile of a public authority from our pilot platform. The format is analogous to (**a**). Here, the dominant culture is Hierarchy, which also emphasizes Internal Focus and Integration. However, Stability and Control take precedence over Flexibility and Discretion. **c** Dominant characteristics, working methods, focus, and success criteria of the four organizational cultures

In this paper, stakeholders refer not to individuals but to organizations. Organizations are characterized by core values, leadership styles, working methods, success criteria, and behavioral norms (Cameron and Quinn 2006). These cultural traits are captured as “Organizational Cultures,” shared among members (Glisson and James 2002), shaping management style (Sulich et al. 2021), decision-making, and member attitudes and behaviors (Smircich 1983), and consisting of enduring beliefs, values, and assumptions (Cameron and Quinn 2006; Schein 2010). The concept of Organizational Culture is supported by the “Competing Values Framework” (Quinn and Rohrbaugh 1983), which organizes organizational values within an integrated, competitive model. This framework is structured along two main dimensions: “flexibility versus stability” and “internal versus external focus.” The intersection of these dimensions forms a matrix with four quadrants representing distinct organizational values (Fig. 2a, b), classifying four cultures: (1) Clan (internally focused and flexible, emphasizing collaboration and unity), (2) Adhocracy (externally focused and flexible, prioritizing innovation and leadership), (3) Market (externally focused and stable, focused on competition and achievement), and (4) Hierarchy (internally focused and stable, emphasizing efficiency and structure) (van Eijnatten et al. 2015). The key characteristics of these four cultures are shown in Fig. 2c.

The concept of Organizational Culture originates from management consulting, where it is used to compare a company’s existing culture with a target culture and to develop strategies for improvement. The Netweave Approach creates Organizational Culture profiles of stakeholders (as shown in Fig. 2a) using the Organizational Culture Assessment Instrument (OCAI). These profiles, along with stakeholder scores on the OCAI subscales (Dominant Characteristics, Organizational Leadership, Management of Employees, Organization Glue, Strategic Emphases, Criteria of Success), are then compared. This combination of culture profiles and subscale scores provides detailed insights into each organization’s working methods, enabling well-founded assumptions about how two stakeholder organizations may interact in collaboration. For example, both the Clan culture of the hunting association in Fig. 2a and the Hierarchy culture of the public authority in Fig. 2b are focused on internal processes and integration, as seen on the x-axis. However, on the y-axis, the Clan culture emphasizes flexibility and discretion, whereas the Hierarchy culture prioritizes stability and control. This divergence could lead to conflicts over the perceived necessity of bureaucratic processes such as project documentation or clear role distributions. As illustrated in Fig. 2c, the Clan culture places significant value on teamwork, trust, and commitment, while the Hierarchy culture emphasizes structure, formality, reliability, and efficiency. These differences could also result in conflicts related to varying project goals or interpersonal aspects of collaboration.

Significant differences in organizational cultures between two collaborating organizations can have a complementary effect or, conversely, result in “working at cross-purposes” (Przesdzink et al. 2024–2). The Netweave Approach uses these insights to anticipate potential areas of conflict and complementarity in collaborative projects. The findings are communicated to stakeholders to foster mutual understanding and ensure that differences are considered in cooperative planning.

### Stakeholder Analysis—Environmental Worldview and Risk Perception

When organizations with an ideological stance for or against nature conservation collaborate with others—or with organizations neutral on the topic—conflicts can arise, regardless of the rational benefits of collaboration (Freese and Rüffer 2005). Therefore, assessing stakeholders’ environmental worldviews may be beneficial to preemptively address such conflicts with tailored communication strategies. The Netweave Approach uses the “New Environmental Paradigm” scale (NEP; Dunlap and Van Liere 1978; German translation by Byrka et al. 2010) to measure these views. The corresponding questionnaire is available in Supplementary Material “Online Questionnaire (OCAI, NEP, MoN)”. The revised NEP encompasses five dimensions: (1) “balance of nature,” (2) “limits to growth,” (3) “anti-anthropocentrism,” (4) “anti-exemptionalism” (regarding human separation from nature), and (5) “possibility of an eco-crisis.” Collectively, high agreement with these dimensions indicates a “pro-ecological worldview” (Dunlap et al. 2000). The NEP’s validity is supported by numerous studies (Davis and Stroink 2016; Dunlap et al. 2000; Hawcroft and Milfont 2010; Pienaar et al. 2015). Communicating differences in NEP scores has been recommended for resolving land use and conservation conflicts (Edgell and Nowell 1989; Kaltenborn et al. 1998; Przesdzink et al. 2024–2).

**Fig. 3:**
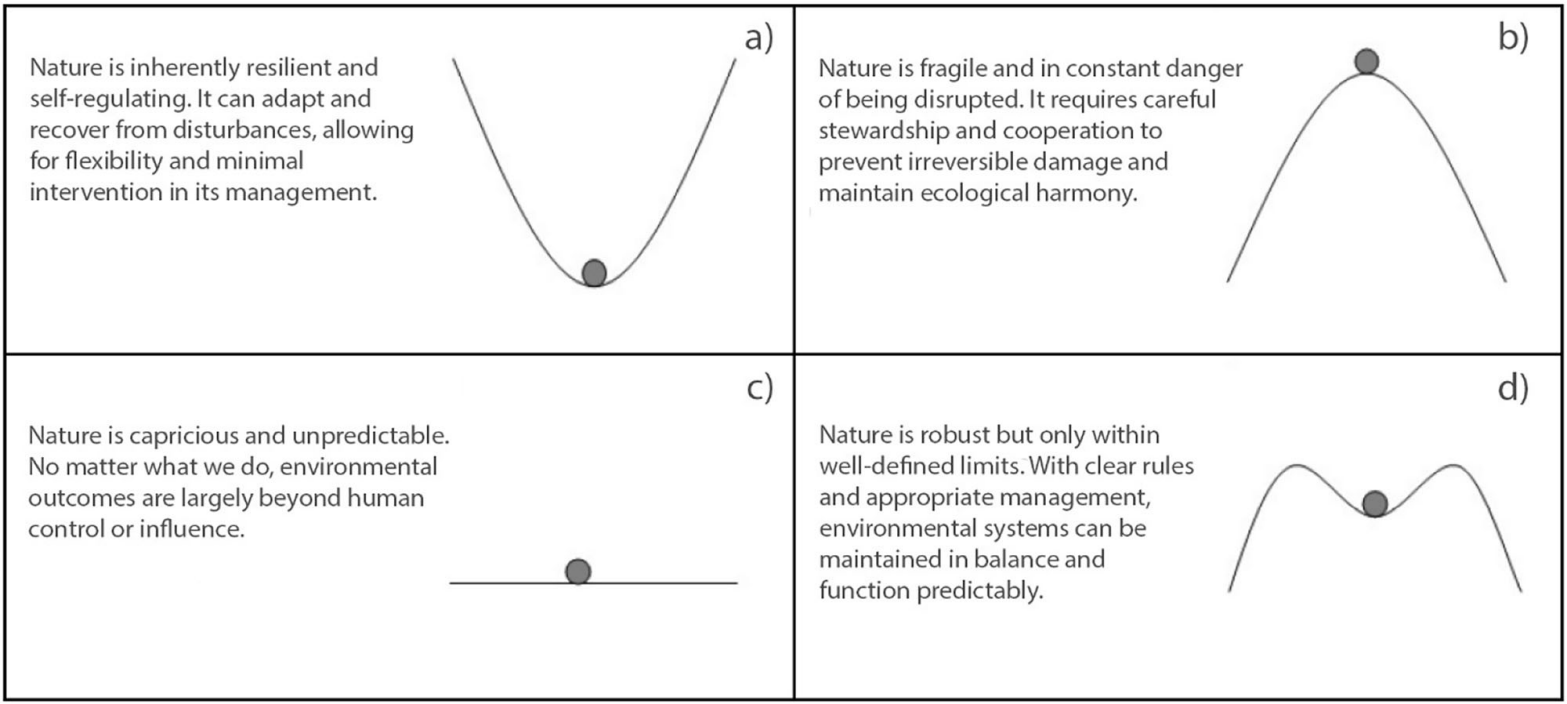
Illustration of the four Myths of Physical Nature (MoN). The gray dot represents “nature,” while the black line indicates its level of fragility. If the dot were to fall off the line, nature would “lose its balance,” leading to ecological catastrophes. **a** Individualistic Myth. **b** Egalitarian Myth**. c** Fatalistic Myth**. d** Hierarchical Myth

The “Cultural Theory” proposes that individuals align with one of four cultural biases, or “myths,” which shape views on human and environmental nature (Schwarz and Thompson 1990). These are the “hierarchical” myth (humans as flawed but governable, nature as resilient within limits), the “egalitarian” myth (humans as cooperative, nature as fragile), the “fatalistic” myth (humans as unreliable, nature as capricious), and the “individualistic” myth (humans as self-centered, nature as benign) (Grendstad and Selle 2000; Schwarz and Thompson 1990). These myths reflect broader worldviews and influence preferences for managing human and environmental systems (van Asselt et al. 1995) and environmental resources specifically (Halik et al. 2022; Van der Wal et al. 2014). Conflicting management preferences often lead to stakeholder disputes (Halik et al. 2018). Since human and environmental biases are not directly correlated, they can be considered independently (Grendstad and Selle 2000). The Netweave Approach focuses solely on the myths of physical nature (MoN), which are more relevant in environmental discussions. The four MoN are illustrated in Fig. 3.

The Netweave Approach compares stakeholders’ NEP scores, NEP subscales, and MoN to evaluate the potential for ideological conflict. If results are similar, ideologically harmonious collaboration is likely. For widely divergent results, differences are communicated to stakeholders beforehand to foster understanding and establish an objective communication basis before joint projects begin.

## Methodology

### Pilot Region

**Fig. 4:**
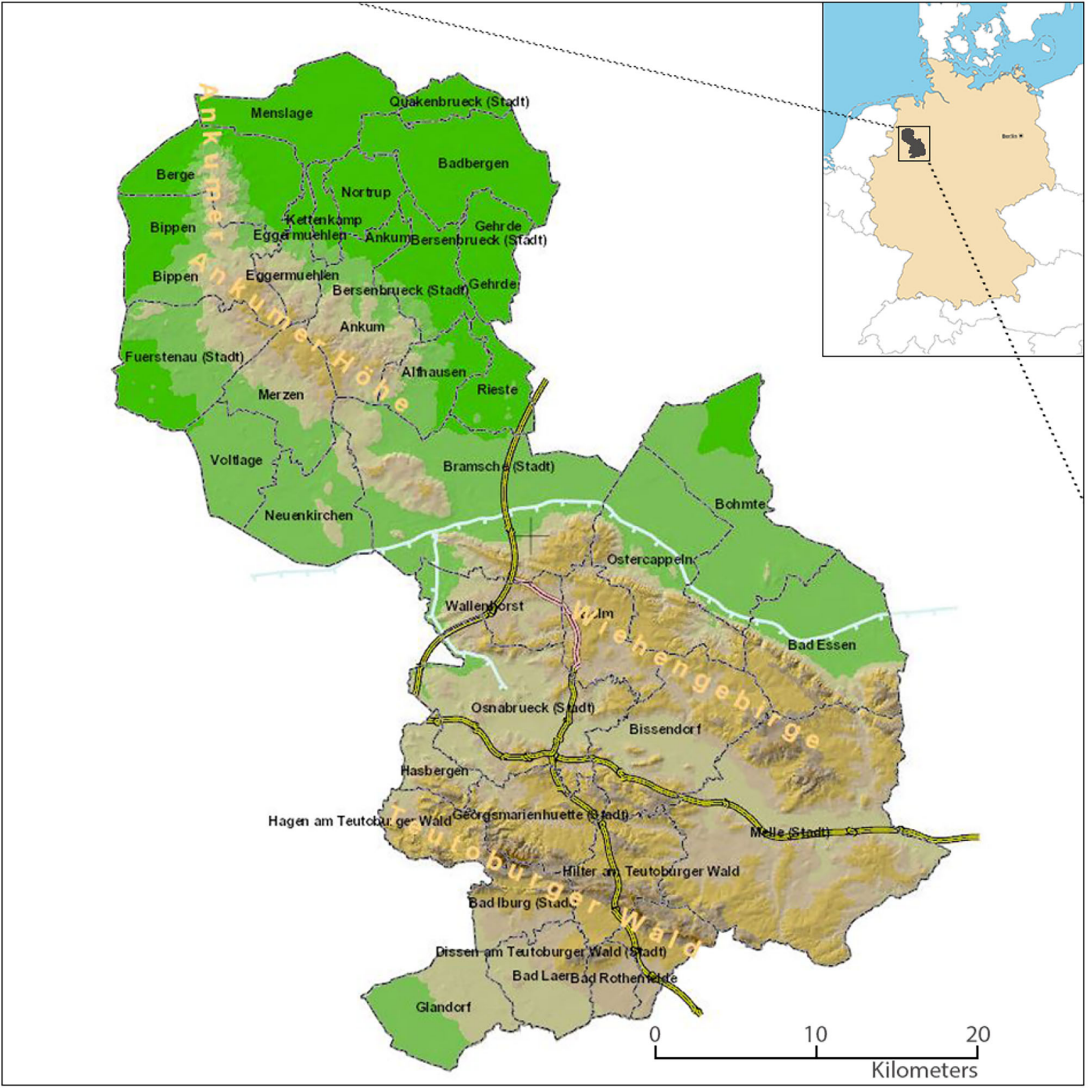
Topographical map of the city and district of Osnabrück. The inset (top right) shows the location of the Osnabrück region within Germany. The brown areas represent the mountain ranges “Ankumer Höhe” (max. 142 m ASL), “Wiehengebirge” (max. 211 m ASL within the region), and “Teutoburger Wald” (max. 331 m ASL within the region). The flat areas of the district, part of the North German Plain in the north and the Münsterland in the south, are marked in green. Municipal boundaries are shown in purple, with municipality names labeled in black. Motorways are highlighted in yellow, and canals in light blue. The small river Hase, which flows through the region from southeast to northwest, and its tributaries are not included. Map of Germany © GingkoMaps-Projekt (http://www.ginkgomaps.com/) under CC-BY-3.0. Map of Osnabrueck provided by Landkreis Osnabrück. Umweltatlas LKOS, geoinfo.lkos.de. 2024. https://geoinfo.lkos.de/webinfo/synserver?project=ua&client=flex

Our pilot region comprises the medium-sized city of Osnabrück, with a population of 171,994 and an area of 120 km² (Melderegister Stadt Osnabrück 2023), where many stakeholders operate in close spatial proximity, and the rural district of Osnabrück, with a population of 366,221 and an area of 2,100 km² (Niedersächsisches Landesamt für Statistik 2021), where stakeholders are more dispersed, and their influence is often limited to their local communities. Figure 4 provides a detailed map of the pilot region.

As of November 2024, our regional database includes 191 stakeholder organizations. These comprise public authorities and academics focused on environmental conservation and resource use, as well as associations from agriculture, water management, fishing, hunting, forestry, apiculture, cultural heritage, environmental education, environmental planning, and conservation. The database expands continuously through a snowball system, where new stakeholders are identified during interviews with existing participants. Therefore, it is inherently dynamic and cannot be considered “complete.”

### Data Acquisition

The data required as part of the Netweave Approach is preferably collected through individual interviews with one, and in some cases two, representatives from each stakeholder organization. Stakeholder organizations in our pilot region were initially identified through an online search for regional organizations in the sectors of environmental research, environmental governance, environmental protection, and the use of regional environmental resources. Additionally, during the data collection interviews, the snowball sampling method was employed to identify further actors relevant to the conservation and sustainable use of regional environmental resources. For a detailed methodology, see Przesdzink et al. (2021). The organizations mentioned during the interviews were subsequently included in the platform as well. The person who is most responsible for communicating with other stakeholders on regional nature conservation issues or who acts as the contact person for such issues, as chosen by the organization, is interviewed in each case. In the case of associations, for example, this could be the chairman, in the case of research groups the group leader or in the case of municipalities the environmental officer.

Data acquisition interviews are conducted in person, online, or via telephone. Online questionnaires on SNA, OCAI, NEP, and MoN (see Supplementary Material “Online Questionnaire (OCAI, NEP, Mon)”), integrated into the platform, are completed either on a tablet provided during in-person interviews or sent to participants via email link. In our case, the highest data quality was achieved during in-person interviews with a tablet, as these facilitated more relaxed communication, particularly with the typically older participants (average age: 54 years, SD = 13.5), who were often less familiar with digital tools. Additionally, the interviewer could assist participants in real time if technical issues arose while completing the questionnaires. For establishing a network consultation system, in-person interviews are recommended to allow stakeholders to personally meet “their” network consultant, demonstrate interest in their concerns, and build trust. However, digital formats are significantly more time-efficient for the consultant, as they eliminate travel time to and from stakeholder locations. In these cases, assisted completion of the questionnaire remains feasible: the interviewer can send the questionnaire link before or during the interview and stay connected with the participant to provide support during the process.

To ensure compliance with data protection regulations, explicit informed consent was obtained from all stakeholders prior to data collection. For the online questionnaires, a consent form was integrated into the beginning of each questionnaire, requiring acceptance before proceeding. For interviews, printed or digital consent forms were provided, which stakeholders signed before the interview began. These consent forms explicitly authorized the storage of stakeholder data, including personal data, in the database and its use by network consultants solely for networking purposes. Access to the data by other stakeholders was not permitted and therefore was not included in the consent process. Similarly, the publication of personal data was neither planned nor requested and is not part of this paper.

During the interview, which typically lasts between 20 and 40 min, questions are asked about the organization’s available and required resources, spatial and thematic limitations of their work, criteria for or against collaborations with other organizations, as well as typical allies and adversaries. The detailed interview guide used is provided in the Supplementary Material “Interview Guideline”. In our case, interviews were digitally recorded and auto-transcribed using Amberscript (Amberscript Global B.V., 2024) or ChatGPT 4.0 (OpenAI, 2024). The collected data, such as information on available resources, were manually entered into the platform based on the transcript. If the network consultation team has fewer resources for data collection, it is also possible to take brief, handwritten notes during the interview and later input these into the platform, or to directly enter the data into the platform during the interview. However, the latter approach may disrupt the natural flow of the conversation.

As the list of stakeholders continuously grows through snowball sampling, it is necessary to have the SNA questionnaire completed at regular intervals to maintain an up-to-date and comprehensive picture of the social network of all stakeholders registered in the platform. This is also advisable for monitoring the development of stakeholder interactions over time. Based on our project experience, resource availability and requirements are dynamic and frequently change. In contrast, an organization’s Organizational Culture, Environmental Worldview, and Environmental Risk Perception tend to remain relatively stable. However, significant changes in these aspects can occur following a management change or internal restructuring. Generally, it is recommended to repeat the entire interview annually, if time allows, to ensure all data remains current.

### Stakeholder Database

**Fig. 5:**
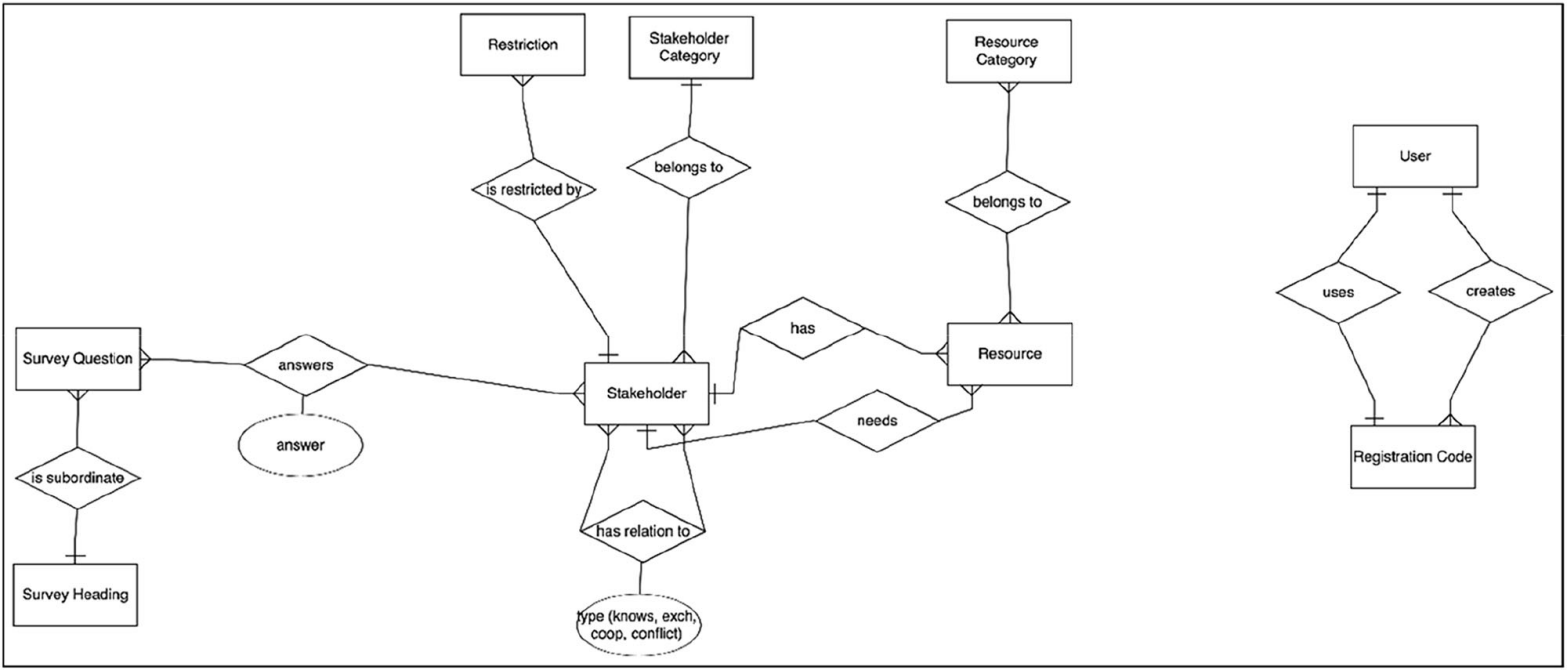
ER Diagram of the Netweave Database Structure. This schematic and simplified representation provides an overview of the entities (rectangles) in the database, their attributes (circles), and their relationships (diamonds)

While the front end of the Netweave platform is presented in detail in the supplementary material “Stakeholder Platform Frontend,” we will focus here on a description of the back end: The backend of the stakeholder database platform was developed using Laravel, a PHP framework, in version 8.12. It operates on PHP 8.0 or higher and uses Blade templates as its server-side templating engine. Blade compiles templates into plain PHP and caches the output for efficiency, avoiding reliance on a separate API layer for front-end interactions. This setup ensures dynamic server-side rendering of the platform’s web interface. The platform utilizes MariaDB as its database management system. The Database scheme (see Fig. 5) is designed to handle stakeholder and resource data efficiently.

Key tables in the Netweave database include the *stakeholders* table, which contains information such as names, contact details, affiliations (via “*category*”), survey participation status, and additional metadata fields related to collaboration criteria and notes. The *resources* table stores details about resources owned or required by stakeholders, with unique identifiers and descriptions. Relationships in the database are represented in different ways, depending on their structure. For 1:1 or 1:n relationships, foreign keys in the related tables are sufficient. For instance, the “*category*” affiliation of a stakeholder is directly stored in the *stakeholders* table using a foreign key. In contrast, n:m relationships or those requiring metadata are represented using pivot tables. Examples include the *stakeholders_have_resources* and *stakeholders_need_resources* tables, which document stakeholder-to-resource relationships, as well as *stakeholder_conflicts_with_stakeholder*, *stakeholder_knows_stakeholder*, *stakeholder_cooperates_with_stakeholder*, and *stakeholder_exchanges_information_with_stakeholder* tables, which handle stakeholder-to-stakeholder interactions. These pivot tables may also store additional information about the relationships, such as a description of how a stakeholder utilizes a specific resource. The tables *questions* and *question_stakeholder* store custom survey questions and stakeholder-specific responses, respectively, facilitating network surveys. Finally, the *users* table contains information on authorized network consultants, including encrypted authentication credentials for accessing the platform.

The backend provides several essential functionalities for the platform’s operation. (1) *Data Management* supports the creation, retrieval, updating, and deletion of stakeholder and resource data, implemented through Laravel controllers. (2) *Survey Integration* allows for the configuration and management of questions related to SNA and evaluations via the database, with stakeholder responses recorded and linked for further analysis. (3) *Recommendation System* functionality identifies stakeholders who can fulfill the resource requirements of others, based on database relationships, offering targeted recommendations.

The platform is hosted on a shared server with specifications including 200 GB of redundant storage, 9 GB of RAM, and a single virtual CPU. It operates under the primary domain netweave.de, with each region served by an independent subdomain (e.g., os.netweave.de for Osnabrueck). These instances are deployed separately but share code managed via a Git repository (https://github.com/Netweave-Managed-Networking). Access to the platform is secured through OAuth2, implemented via Laravel Sanctum. Stakeholder data is encrypted using OpenSSL with AES-256 and AES-128 algorithms, ensuring data confidentiality. Only network consultants have direct access to the system, with sensitive information appropriately restricted.

The platform’s architecture is designed for scalability, allowing the straightforward addition of new regions by duplicating backend instances. Each instance operates independently to maintain data separation between regions.

### Network Consultancy

The networking consultation involves five primary use cases. (1) *Addressing Resource Requirements*: The first use case focuses on fulfilling resource requirements already listed in the platform. This process considers the automatic, resource-based networking recommendations within a stakeholder’s profile (Supplementary Material “Stakeholder Platform Frontend”; Fig. 3b) and involves manually compiling a list of stakeholders who can meet these requirements. The manual process evaluates organizational culture, NEP scores, MoN preferences, spatial and thematic limitations, and personal demands regarding collaboration quality. Additionally, the profile owner’s social network is reviewed to analyze their current interactions with potential partners. Existing collaboration is optimal, prior acquaintance is advantageous as no introduction is needed, and conflict is problematic due to prior negative experiences. Based on these factors, an informed recommendation is made regarding the best-fit stakeholder organization. This step requires practical experience with the platform. In the long term, the goal is to integrate all data into the automatic recommendations to simplify the process.

(2) *Adding a New Stakeholder Organization to the Platform:* The second use case involves onboarding a new stakeholder organization. On the stakeholder list (Supplementary Material “Stakeholder Platform Frontend”; Fig. 5), accessible via the button in the top left corner of the main menu (Supplementary Material “Stakeholder Platform Frontend”; Fig. 2), a new profile is created and named. Contact information can already be manually entered. The organization’s representative is sent a link to the landing page (Supplementary Material “Stakeholder Platform Frontend”; Fig. 1), where their organization is automatically listed. By selecting their organization, the representative can complete all active online questionnaires for their organization. Additional data collected during interviews are manually entered into the profile by the network consultant.

(3) *Responding to New Resource Requests*: The third use case occurs when stakeholders personally report new resource requirements or when someone without a profile in the platform submits a quick request for a resource. In this scenario, the search function (Supplementary Material “Stakeholder Platform Frontend”; Fig. 4) is used to compile a list of stakeholders who can meet the requirement. If the requesting stakeholder is already in the database, the manual process from the first use case is followed. If the stakeholder organization is not in the database, in our case they must first get a profile by following the second use case. Afterward, the process again follows the first use case. Whether to handle external requests or require stakeholders to create a profile before being advised should be determined during the initial platform setup. Since stakeholders are often motivated to offer resources to others in anticipation of receiving resources later, a closed system requiring stakeholders to have a profile and provide resources might be preferable. This approach could prevent “free-riders” who only make requests without contributing resources themselves.

(4) *Compiling Stakeholders for a Major Project*: The fourth use case involves assembling relevant stakeholders for a large-scale project. Using the search function, stakeholders possessing the required resources, competencies, and areas of responsibility are identified. Their profiles are manually compared, considering socio-psychological factors. An informed assessment identifies potential conflicts, complementary capabilities, and stakeholders likely to collaborate effectively. Project communication and meeting moderation are aligned accordingly, with commonalities and differences made transparent. Shared guidelines for collaboration are established to ensure a cohesive working relationship.

(5) *Viewing the Social Network of All Stakeholder Organizations*: Using the button in the top right corner of the main menu (Supplementary Material “Stakeholder Platform Frontend”; Fig. 4), cross-tables of interactions among stakeholder organizations across the recorded dimensions—Acquaintance, Collaboration, and Conflict—can be downloaded. These interactions can be visualized in a network graph (see Fig. 1) using freeware tools such as Gephi. Such a graph is particularly useful for providing an overview of the overall network structure or identifying distinct, separated groups. Additionally, it allows stakeholders to see their own position within the network. If desired, the cross-tables can also be utilized for more advanced network analyses.

### Specific Application Example

The concept of networking recommendations is illustrated below using a real-life example of Use Case 1: Addressing Resource Requirements. The stakeholder in this case was a public authority seeking biological expertise for the creation of a meadow orchard. A database analysis identified three stakeholders capable of meeting this need. Further examination revealed that the public authority’s available resources could provide reciprocal value to all three stakeholders, creating potential win-win scenarios. Table 1 summarizes the following process of manually assessing additional platform criteria to determine the best fit for collaboration.

**Table 1:** Comparison of three stakeholders (A, B, and C) regarding their fit with the Netweave Approach criteria for a stakeholder with resource requirements

Matching Aspect	Stakeholder A	Stakeholder B	Stakeholder C
Organizational Culture	o	-	+
Environmental Worldview	+	+	o
Myth of Nature	o	+	+
Interaction in Social Network	Is known	Is known	Unknown
Spatial fit	-	+	+
Thematic fit	+	+	o
Personal demands	+	+	o

Ratings are + (“favorable”), o (“neutral”), and – (“unfavorable”), assigned subjectively by the network consultant relative to the other stakeholders rather than as absolute values. Interactions in the social network reflect existing relationships between the public authority and the other stakeholders. Despite differences in organizational culture, Stakeholder B emerged as the most suitable partner and was recommended for collaboration

To assess compatibility, we analyzed differences in Organizational Cultures, Environmental Worldviews, and MoN between the public authority and the three stakeholders. Stakeholder B showed the largest divergence in organizational culture but the smallest ideological differences. Stakeholders A and B had NEP scores closely aligned with the public authority, while Stakeholder C showed significant deviation. Regarding MoN, Stakeholders B and C shared the public authority’s belief, while Stakeholder A did not.

The social network review showed that Stakeholders A and B had the best relationships with the public authority. Spatial and thematic constraints were then evaluated. Stakeholder A was geographically distant, while B and C were located nearby. However, Stakeholder C was only marginally involved in meadow orchard projects, making them thematically the least suitable. Lastly, personal collaboration criteria were reviewed. Stakeholder C expressed skepticism towards bureaucracy, making them incompatible with the hierarchical public authority. Overall, Stakeholder B, a nature conservation association, was determined to be the best fit. The public authority could reciprocate by offering financial support.

After confirming compatibility, we contacted the public authority to obtain consent for networking advice. This was followed by a call with the nature conservation association to confirm their agreement and resource availability. With positive responses from both sides, we sent a joint email to initiate collaboration. To address cultural differences, the proposal highlighted key considerations: The public authority’s hierarchical culture emphasizes regulated processes, which the association was advised to accommodate when providing orchard advice or writing funding applications. The public authority was encouraged to be flexible if the association’s approach did not fully align with its expectations. Simultaneously, the association’s clan culture, prioritizing personal and collegial communication, was acknowledged, and the public authority was asked to engage accordingly. Both parties were advised to show mutual understanding during the collaboration. This tailored approach facilitated the successful initiation of collaboration between the stakeholders.

### Toward an Integrated Compatibility Framework for Stakeholder Matching

Based on the Netweave platform’s theoretical constructs we developed an integrative framework to quantify the compatibility of stakeholder organizations and provide systematic recommendations for collaboration. This framework responds to the need for a cohesive model that brings together the diverse dimensions assessed in the Netweave Approach. It is designed to support manual consultancy using the current version of the Netweave platform and, in future platform-versions, automated stakeholder matching.

**Fig. 6:**
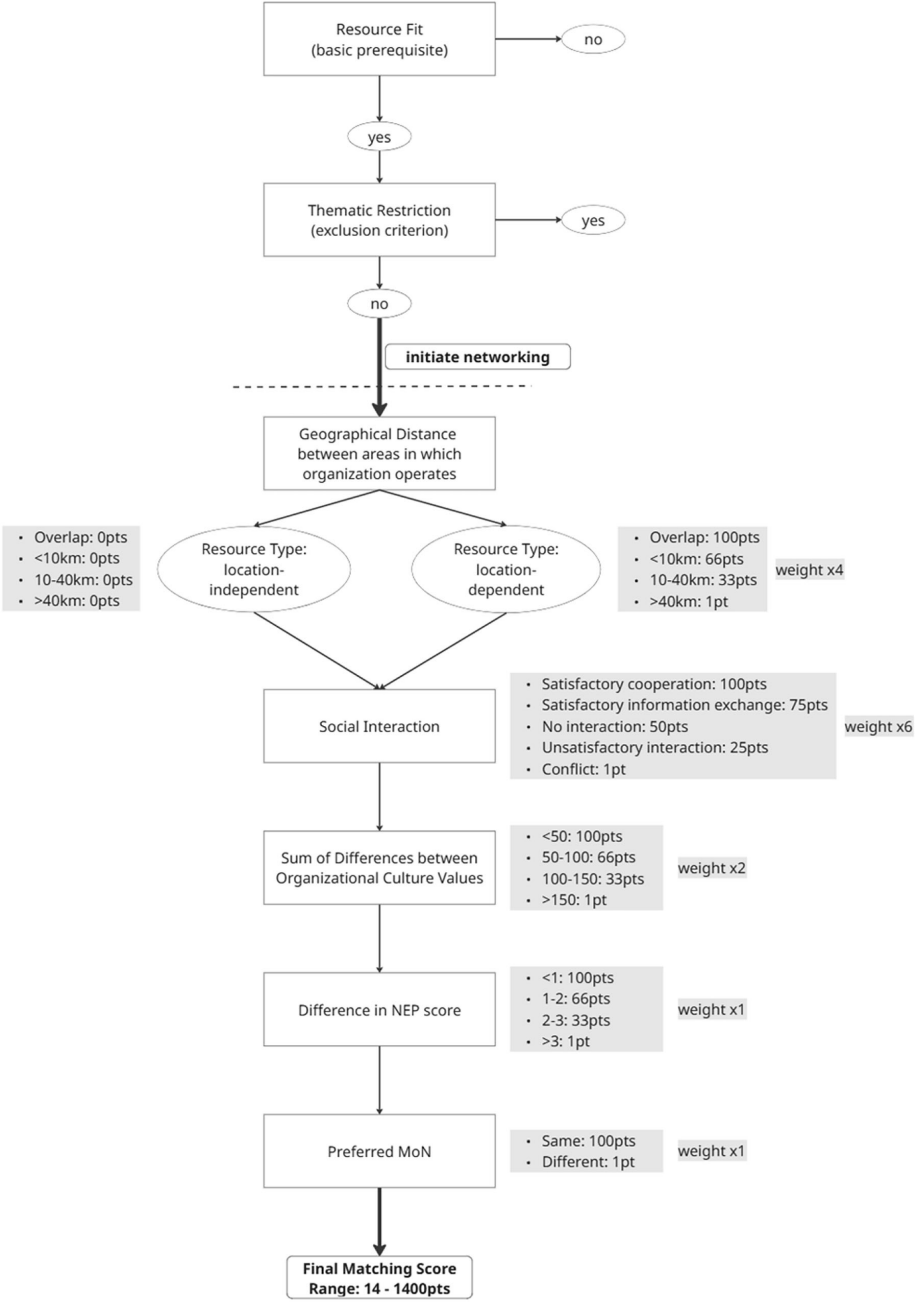
Logic model for calculating the Matching Score between two stakeholders. The model considers compatibility across seven dimensions: resource fit, thematic restrictions, geographical proximity (only if location-dependent), social interaction history, organizational culture similarity, alignment in environmental worldviews (NEP), and shared perceptions of nature (MoN). Each variable contributes to a weighted score ranging from 14 (low compatibility) to 1400 points (very high compatibility)

The logic model (Fig. 6) calculates a *Matching Score* ranging from 14 to 1400 points, providing a numeric indicator of collaboration potential. The weighting of the seven dimensions in the Matching Score reflects their relative operational relevance for predicting successful collaboration, as derived from empirical findings in our pilot region and from theoretical considerations:

**1. Resource Fit** is treated as a strict prerequisite: Only stakeholder pairs for which at least one actor provides a resource that the other requires are considered for collaboration. Ideally, both organizations benefit through reciprocal resource exchange. This restriction reflects findings from interviews and empirical studies in our pilot region, where time constraints led many stakeholders to reject networking offers without clear, direct benefit (Przesdzink et al. 2021; Przesdzink et al. 2024–2).

**2. Thematic Restrictions** are considered exclusion criteria, as they directly reflect a stakeholder’s declared unwillingness to collaborate outside a defined scope. For instance, if one stakeholder explicitly limits cooperation to research-related projects, while another seeks support in a non-research context, acceptance of a collaboration by the first is highly unlikely and even an otherwise strong match would be excluded.

**3. Geographical Distance** is weighted strongly (weight 4) for location-dependent resources (e.g., land, equipment, or on-site services). It replaces the measure *spatial fit* used in our pilot platform, which was a rather vague free-text field, with a more finely structured and measurable variable. These distances are considered relevant because spatial proximity was a frequent criterion for collaboration preference in our pilot region. In contrast, for location-independent resources (e.g., data analysis, policy advice), distance does not affect the score. The specific thresholds (e.g., <10 km, 10–40 km) were derived from our observations in a mid-sized German district and may need to be adjusted for more local or national contexts. In the current framework, we propose using Euclidean distance due to its computational simplicity and ease of integration into existing systems. However, in future versions of the platform, more precise isochrone-based measures—which take into account travel time and infrastructure—may offer a more realistic assessment of accessibility and collaboration feasibility (Neutens et al. 2010).

**4. Social interaction history** is assigned the highest weight (weight = 6) because it provides a direct, empirical record of how two stakeholders have already interacted in practice. Positive past collaboration or information exchange serves as concrete evidence that productive cooperation is feasible, while past conflicts contradict this assumption—evidence that implicitly integrates the influence of many other factors that shape relationships, such as political alignment or opposition, personal animosities, group memberships, age, socialization, educational background, and other socio-cultural variables. In this sense, interaction history functions as an “outcome-based” indicator that encapsulates both measurable and unmeasured determinants of compatibility. In contrast, psychological constructs such as organizational culture, environmental worldview, and preferred myths of nature capture only specific aspects of this broader set of influences.

**5. Organizational Culture Fit** is assessed by calculating the sum of differences across the six OCAI dimensions. Lower divergence implies better compatibility in work styles and communication habits (Przesdzink et al. 2024–1). This dimension is weighted with a factor of 2, as substantial differences in working styles, communication habits, and decision-making norms can cause friction even when practical incentives for collaboration exist. Nevertheless, culture is only one of many potential compatibility factors and does not carry the same integrative, outcome-based evidence as social interaction history.

**6. Ideological Alignment** is reflected in two separate but related constructs: the *difference in NEP scores* and agreement in *preferred Myths of Nature* (MoN). Both reflect the organization’s general stance toward environmental protection and sustainability. Given their conceptual similarity, both were included with weight 1 each to combined match the weight of organizational culture fit. While ideological alignment can support trust-building and reduce conflict risk, misalignment does not necessarily prevent collaboration if other drivers—such as resource interdependence or political necessity—are strong.

This weighting scheme ensures that the most decisive, empirically grounded predictor—interaction history—has the largest influence, while psychological and ideological variables are integrated in a way that balances their theoretical importance with their more limited predictive scope. Spatial and thematic constraints, where applicable, act as strong contextual filters, and resource fit remains the fundamental condition for considering any match. While the resulting score is continuous, preliminary thresholds can provide practical guidance for network consultants. Based on the theoretical range of 14–1400 points and our pilot experience, the following intervals may serve as a tentative orientation: scores above 1000 indicate very high compatibility and can be acted upon with minimal moderation needs; scores between 700 and 1000 suggest high compatibility, with only minor preparatory communication recommended; scores in the range of 400–700 represent medium compatibility, where targeted moderation or pre-collaboration dialog is advisable; scores between 200 and 400 indicate low compatibility, and collaboration should be initiated only if there is strong external or political necessity, accompanied by careful facilitation; and scores below 200 imply very low compatibility, where active matchmaking is generally not recommended. These intervals are intended as starting points for operational decision-making and should be refined over time using empirical data from specific network contexts.

In cases where major differences in environmental worldview or organizational culture are detected, these aspects are addressed selectively and with care during the consultancy process. A brief explanation of the divergence and the other party’s perspective is provided to support mutual understanding. This situational communication offers clear benefits: it can prevent misunderstandings, prepare stakeholders for differing expectations, and help build trust. However, it also bears risks—particularly if such information is perceived as judgmental or overly theoretical. For this reason, we limit such exchanges to cases where ideological or cultural distance is likely to affect collaboration, and frame them as neutral context information rather than evaluation.

Importantly, we deliberately chose not to calibrate the threshold values of organizational culture differences and NEP-score differences (e.g., for “high” or “low” NEP divergence) to our own network’s internal distribution, in order to maintain absolute compatibility thresholds. While in our pilot region the cultural and ideological diversity among stakeholders was limited, normalizing based on local variance would have overstated the importance of small differences in relatively homogeneous systems. Instead, the current scaling ensures that in such homogeneous networks, ideological or cultural mismatches have minor impact on the score, which is consistent with stakeholder feedback. It also simplifies the case-independent use of the framework.

### The Potential Role of Structural and Spatial Network Metrics

While the Netweave platform already integrates stakeholder interaction data across various relational dimensions—such as acquaintance, collaboration, and conflict—classical social network metrics like degree centrality, betweenness centrality, or modularity have not been systematically applied within the operational consultancy process. In earlier stages of the project, however, such metrics were used to identify scale-crossing brokers, structural communities, and network hubs in the Osnabrück region (Przesdzink et al. 2021). Although these analyses provided valuable scientific insights, their practical relevance for everyday network consultancy proved limited.

During interviews and UX workshops, stakeholder organizations consistently emphasized severe time constraints as a central barrier to networking engagement. As a result, we prioritized direct resource-based matchmaking over more abstract structural considerations. Moreover, network metrics such as centrality or modularity, though well-established in sociological research, were often perceived as too complex or abstract by regional network managers without a background in SNA. Consequently, these metrics were not included in the platform’s operational layer.

Nonetheless, integrating structural network measures remains promising—especially in the context of broader consensus-building processes or future research applications. Metrics such as degree centrality can help identify influential actors, betweenness centrality highlights bridge-builders, and modularity can detect stakeholder clusters requiring targeted inclusion strategies (Bodin and Crona 2009; Bodin et al. 2017; Prell et al. 2009). While not directly relevant to everyday platform use, these analyses can be done using the Adjacency Matrices provided by the platform and may support more strategic interventions or contribute to academic understanding of stakeholder systems.

In addition, spatial proximity has so far only been considered as a qualitative element (spatial fit) based on stakeholder self-reports. The integrative framework presented in this paper now proposes a more systematic use of geographical distance—e.g., through threshold values for location-dependent resources—as part of the matching algorithm. Further enhancements such as spatial network visualizations or GIS-based proximity measures (e.g., Isochrones) could be explored in future developments of the platform.

## Limitations and Improvement Suggestions

In addition to the specific weaknesses highlighted in the Supplemetary Material “Implementation Analysis”, this section discusses general methodological shortcomings and proposes potential improvements.

As stakeholder data were collected via expert interviews, it is important to acknowledge the potential for response bias. Interviewees may aim to present themselves positively, aligning their answers with perceived interviewer expectations—a phenomenon rooted in the concept of social desirability (Bogner and Landrock 2015). Consequently, the accuracy and honesty of responses may be affected. Furthermore, interviewees might not fully represent the interests and views of the organizations they represent. Meuser and Nagel (2009) note the difficulty in separating the individual’s role as a representative of the organization from their personal identity. To minimize this issue, interviews were conducted exclusively with individuals in managerial positions or designated contact persons, as they likely possess a comprehensive understanding of their organization’s objectives and activities. These individuals are also the ones most likely to engage with other stakeholders during collaborations. While Cameron and Quinn (2006) suggest that organizations may consist of distinct subcultures, these subcultures generally share core elements of the overarching organizational culture. Similarly, Friedkin (1998) asserts that individual attitudes and opinions often reflect those of surrounding members, supporting the expectation that organizational members share core values assessed by our questionnaires (Przesdzink et al. 2024–2).

The German version of the OCAI used in this study was developed through the translation-backtranslation method (Brislin 1970) but was not separately validated before the project. Additionally, the ipsative rating scale posed challenges, as participants struggled to allocate 100 points across four statements, often due to either misreading the instructions or calculation errors (Przesdzink et al. 2024–2). In our platform, these values were adjusted using ratio calculations, which deviates from the original OCAI methodology. A potential alternative could involve replacing the forced-choice format with Likert scales, asking participants to rate their perceived identification with each cultural statement. However, this might reduce the accuracy of the assessment.

An automated solution, such as a “dating app” for stakeholders, could suggest collaboration partners based on complementary resources and socio-psychological compatibility. However, the adoption of such a digital tool requires a certain level of affinity for digital solutions, which, as discussed, is currently lacking in our region. Nevertheless, as digitalization progresses, it is likely that the stakeholder landscape will eventually be ready for such a solution. This could reduce the manual workload associated with stakeholder matchmaking and increase the scalability of the Netweave Approach.

## Transferability

In general, these are regions and topics where stakeholder interactions impair conservation or sustainability in general and where stakeholder conflicts may have grave consequences for ecosystems management or Sustainable Development. One use case are large-scale protected areas such as nature parks or biosphere reserves, as they generally have in common that nature conservation must be reconciled with land use stakeholders and tourism, which often leads to stakeholder conflicts (Sinthumule et al. 2020; Steinhäußer et al. 2015; Von Ruschkowski and Mayer 2011; Young et al. 2005; Yuxi et al. 2022).

### Biosphere Reserves and Nature Parks

Biosphere reserves and Nature Parks represent optimal application examples for the Netweave Approach. Biosphere reserves, by definition, fulfill three core functions: (1) biodiversity conservation, (2) promotion of sustainable social and economic development, and (3) research, monitoring, and education (UNESCO 1996). Biosphere reserves explicitly include anthropogenic use of their areas and ecosystems (Weber and Weber 2014). Consequently, they must balance the interests of various stakeholders with environmental conservation, ensuring that both people and nature thrive without one disadvantaging the other (Van Cuong et al. 2017). This necessitates collecting stakeholder data and highlighting the importance of collaboration among stakeholders from diverse backgrounds, often with conflicting objectives. The literature identifies several requirements for successful biosphere reserves, including effective management, adequate finances and resources, clear communication, and stakeholder collaboration and participation (Bouamrane et al. 2016; Van Cuong et al. 2017; Schliep and Stoll-Kleemann 2010; Schultz et al. 2011). Among these, stakeholder collaboration and participation are deemed the most critical factors (Van Cuong et al. 2017). Given their dual focus on nature and people, biosphere reserves present a compelling use case for the Netweave Approach, which provides insights into stakeholders’ characteristics, resources, and relationships.

The tasks of German “Nature Parks” include nature conservation, tourism, environmental education, and landscape maintenance (Weber and Weber 2014). Additionally, they are legally obligated to promote sustainable regional development. Weber and Weber (2014) emphasize that Nature Parks should evolve into regional managers tasked with coordination and networking, engaging local actors, supporting projects, and resolving disputes among stakeholders. The Netweave Approach can serve as a tool to facilitate this transformation, supporting a bottom-up process of sustainable regional development, as advocated by Weber and Weber (2014).

### Application in Other Fields of Sustainable Development

A stakeholder database can also be valuable beyond the specific context of large protected areas. Since effective environmental management should extend beyond protected areas to the broader landscape—for example, to enhance habitat connectivity (Noss 1991; Tambosi et al. 2014)—establishing stakeholder networks can contribute significantly to the sustainable development of regions, particularly those with a low proportion of protected areas. However, a central institution is required to host the network consultancy, as this role is not inherently provided—unlike in large protected areas where management is typically well-established. This role could be fulfilled by regional authorities or larger associations. As outlined in detail in the Supplementary Material “Implementation Analysis”, it is crucial that the institution chosen is regionally recognized and perceived as competent, trustworthy, and impartial. Furthermore, they must be able to ensure the long-term maintenance and support of the platform to guarantee its effectiveness.

Since a Netweave platform is essentially a tool for data-driven and efficient networking of organizations, it has the potential to be applied in entirely different contexts. Use cases that have already emerged from presentations of the Netweave Approach at conferences include regional climate protection networks, national networks for advancing the energy transition in heating systems, and networking booths at a major environmental trade fair. With adjustments to data structure and stakeholder types, the approach may be applicable in contexts beyond nature conservation.

### Prerequisites for Setting Up New Use Cases

For organizations or network managers interested in exploring the Netweave Approach in their own context, we provide a freely accessible technical guide and sample configuration materials at: https://github.com/Netweave-Managed-Networking (open access). These materials outline the platform’s technical setup and database structure and are intended to support the deployment of independent Netweave instances. While the conceptual foundations are detailed in this paper, the practical application of the approach in new contexts generally requires case-specific adaptation—for instance, identifying relevant stakeholder groups, selecting or modifying assessed constructs, or aligning processes to the institutional environment. We anticipate some of these adjustments below and discuss which variables may require contextualization. We welcome interest in applying the Netweave Approach to new network settings and are happy to support implementation efforts where possible—either through advisory input or collaborative development. Interested actors are encouraged to get in touch via the contact information provided in the repository.

The set of stakeholders used in our use case reflects the administrative and voluntary structures in northern Germany. Transferability to other regions in Western Europe should be relatively seamless, provided similar administrative structures are present. Nonetheless, it is essential to develop an adapted stakeholder set in collaboration with regional key players at the beginning of any new implementation. This stakeholder set can then be expanded during data collection via a snowball sampling system to include all regionally relevant stakeholders over time. In regions with culturally or administratively distinct contexts, such as those on other continents, stakeholder sets may differ significantly from ours.

Furthermore, the psychological constructs used in the Netweave Approach—Organizational Culture, Environmental Worldview, and Environmental Risk Perception—have primarily been validated in Western societies. Their applicability outside this context is uncertain (Abi-Raad 2019; Corral-Verdugo and Armendariz 2000; Ogunbode 2013; Przesdzink et al. 2024–2). When applying the platform in culturally or administratively distinct regions, both the stakeholder composition and the constructs being assessed must be carefully reviewed and, if necessary, adapted. This adaptation is technically straightforward, as the platform’s questionnaires are fully editable. For instance, if constructs more relevant to indigenous communities in South America are identified, the questionnaires can be adjusted accordingly while still enabling automated data collection. In remote regions without internet access, data collection via paper-and-pencil questionnaires during stakeholder interviews is a viable alternative. These responses can subsequently be manually entered into the platform.

In other use cases, it is crucial to handle data protection with the same level of care, adhering to relevant guidelines, such as the GDPR (General Data Protection Regulation) in Germany. All types of data collected and their intended uses should be explicitly included in the consent process. This includes potential future uses, as retroactively adding new purposes would require obtaining new consent from all stakeholders—a time-consuming and cumbersome process for all involved. A sample GDPR-compliant privacy statement is included in the Supplementary Material “Consent Form for Data Collection and Use” as a reference.

To implement the Netweave Approach in a new use case, network consultants must be trained in its methodology. These individuals should possess strong communication skills, be fluent in the regional language and dialect, and ideally come from the region to build trust and rapport with stakeholders—prerequisites for effective stakeholder management (Gorris and Koch 2024; Torabi et al. 2016). In contexts involving indigenous communities, such as those in Central and South America, building trust often requires significant time and effort. For example, researchers may need to live with the community for a month or more to establish relationships before conducting interviews and gathering information (O’Brien et al. 2022; Riley and Kaneakua 2024). This process should be explicitly planned and integrated into project timelines to ensure meaningful engagement. At the same time, network consultants must maintain neutrality, avoiding excessive alignment with specific stakeholders. An interdisciplinary background, such as a degree in biology and agricultural sciences, can further enhance their effectiveness by providing relevant expertise for stakeholder consultations.

It is important to emphasize that the stakeholder database serves as a foundational tool for interventions, but the success of these interventions depends on the skills of the network consultants in the region. If these individuals can implement the recommendations of the networking consultation and foster co-creation processes (Wielinga and Robijn 2020) among stakeholders, this approach has the potential to reduce conflicts and enhance both the efficiency and public acceptance of nature conservation and sustainable development efforts.

## Conclusion

The Netweave Approach offers an innovative solution to the challenges of stakeholder collaboration in conservation and sustainable development. By integrating SNA, resource mapping, and psychological assessments of organizational cultures and environmental worldviews, it provides a comprehensive framework for understanding and facilitating interactions among diverse stakeholders. The pilot implementation in Osnabrück demonstrated the potential of this approach to foster effective and mutually beneficial collaborations, while also highlighting areas for improvement, such as ensuring cultural compatibility and addressing data collection challenges.

The adaptability of the Netweave platform to other contexts, from biosphere reserves to regional climate initiatives, underscores its versatility. However, its successful deployment requires careful consideration of regional administrative structures, cultural contexts, and the training of dedicated network consultants. The findings from this study emphasize the importance of trust-building, local expertise, and adherence to data protection standards to ensure stakeholder engagement and long-term sustainability.

Future developments, such as automated compatibility assessments and tailored digital tools, could further enhance the efficiency and scalability of the approach. By bridging gaps in communication and collaboration, the Netweave Approach has the potential to significantly improve the management of ecological and socio-environmental challenges, fostering both ecological resilience and stakeholder inclusion across different governance and landscape contexts.

## Supplementary information

Consent Form for Data Collection and Use

Implementation Analysis

Interview Guideline

List of Resource Categories

Online Questionnaire (OCAI, NEP, MoN)

Stakeholder Platform Frontend

## Data Availability

No datasets were generated or analyzed during the current study.

## References

[CR1] Abi-Raad M (2019) Western organizational theories: middle eastern style: how much do you know about the culture. J Organ Manag Stud 2019:1–16. 10.5171/2019.730213

[CR2] Amberscript Global B.V. (2024) Amberscript [Software]. https://www.amberscript.com

[CR3] Ansong M, Røskaft E (2011) Determinants of attitudes of primary stakeholders towards forest conservation management: a case study of Subri Forest Reserve, Ghana. Int J Biodivers Sci, Ecosyst Serv Manag 7(2):98–107. 10.1080/21513732.2011.613411

[CR5] Balmford A, Cowling RM (2006) Fusion or failure? The future of conservation biology. Conserv Biol 20(3):692–695. https://www.jstor.org/stable/3879233 16909557 10.1111/j.1523-1739.2006.00434.x

[CR6] Bastian M, Heymann S, Jacomy M (2009) Gephi: an open source software for exploring and manipulating networks. International AAAI Conference on Weblogs and Social Media. 10.1609/icwsm.v3i1.13937

[CR7] Bazzoli GJ, Casey E, Alexander JA, Conrad DA, Shortell SM, Sofaer S, HasnainWynia R, Zukoski AP (2003) Collaborative initiatives: where the rubber meets the road in community partnerships. Med Care Res Rev 60(4_suppl):63S94S. 10.1177/1077558703259082 10.1177/1077558703259082 14687430

[CR8] Bendtsen EB, Clausen LPW, Hansen SF (2021) A review of the state-of-the-art for stakeholder analysis with regard to environmental management and regulation. J Environ Manag 279: 111773. 10.1016/j.jenvman.2020.111773 10.1016/j.jenvman.2020.111773 33310243

[CR9] Bennett NJ, Roth R, Klain SC, Chan K, Christie P, Clark DA, Wyborn C (2017) Conservation social science: understanding and integrating human dimensions to improve conservation. Biol Conserv 205:93–108. 10.1016/j.biocon.2016.10.006

[CR10] Biggs D, Abel N, Knight AT, Leitch A, Langston A, Ban NC (2011) The implementation crisis in conservation planning: could “mental models” help?. Conserv Lett 4(3):169–183. 10.1111/j.1755-263X.2011.00170.x

[CR11] Blackman A (2009) Colombia’s discharge fee program: Incentives for polluters or regulators?. J Environ Manag 90(1):101–119. 10.1016/j.jenvman.2007.08.010 10.1016/j.jenvman.2007.08.010 18086514

[CR12] Bodin Ö, Crona BI (2009) The role of social networks in natural resource governance: what relational patterns make a difference?. Glob Environ Chang. 19(3):366–374. 10.1016/j.gloenvcha.2009.05.002

[CR13] Bodin Ö, Sandström A, Crona B (2017) Collaborative networks for effective ecosystem-based management: a set of working hypotheses. Policy Stud J 45(2):289–314. 10.1111/psj.12146

[CR107] Bogner K, Landrock U (2015) Antworttendenzen in standardisierten Umfragen. GESIS–Leibniz Institut für Sozialwissenschaften. 10.15465/gesis-sg_016

[CR14] Boschet C, Rambonilaza T (2018) Collaborative environmental governance and transaction costs in partnerships: evidence from a social network approach to water management in France. J Environ Plan Manag 61(1):105–123. 10.1080/09640568.2017.1290589

[CR15] Bouamrane M, Spierenburg M, Agrawal A, Boureima A, Cormier-Salem MC, Etienne M, … & Mathevet R (2016) Stakeholder engagement and biodiversity conservation challenges in social-ecological systems: some insights from biosphere reserves in western Africa and France. Ecol Soc 21(4). https://www.jstor.org/stable/26270009

[CR16] Brislin RW (1970) Back-translation for cross-cultural research. J Cross-Cult Psychol 1(3):185–216. 10.1177/135910457000100301

[CR17] Byrka K, Hartig T, Kaiser FG (2010) Environmental attitude as a mediator of the relationship between psychological restoration in nature and self-reported ecological behavior. Psychol Rep 107(3):847–859. 10.2466/07.PR0.107.6.847-859 21323143 10.2466/07.PR0.107.6.847-859

[CR18] Cameron, KS, Quinn, RE (2006) Diagnosing and changing organizational culture—based on the competing values framework. Jossey-Bass

[CR20] Cohen PJ, Evans LS, Mills M (2012) Social networks supporting governance of coastal ecosystems in Solomon Islands. Conserv Lett 5(5):376–386. 10.1111/j.1755-263X.2012.00255.x

[CR21] Corral-Verdugo V, Armendariz LI (2000) The “new environmental paradigm” in a Mexican community. J Environ Educ 31(3):25–31. 10.1080/00958960009598642

[CR22] Crow DA, Baysha O (2013) “Conservation” as a catalyst for conflict: considering stakeholder understanding in policy making. Rev Policy Res 30(3):302–320. 10.1111/ropr.12020

[CR23] Davis AC, Stroink ML (2016) The relationship between systems thinking and the new ecological paradigm. Syst Res Behav Sci 33(4):575–586. 10.1002/sres.2371

[CR24] Díaz SM, Settele J, Brondízio E, Ngo H, Guèze M, Agard J, … & Zayas C (2019) Global assessment report on biodiversity and ecosystem services of the Intergovernmental Science-Policy Platform on Biodiversity and Ecosystem Services. IPBES. 10.5281/zenodo.6417333

[CR25] Dunlap RE, Van Liere KD (1978) The “new environmental paradigm”. J Environ Educ 9(4):10–19. 10.1080/00958964.1978.10801875

[CR26] Dunlap RE, Van Liere KD, Mertig AG, Jones RE (2000) New trends in measuring environmental attitudes: measuring endorsement of the new ecological paradigm: a revised NEP scale. J Soc Issues 56(3):425–442. 10.1111/0022-4537.00176

[CR28] Edgell MC, Nowell DE (1989) The new environmental paradigm scale: wildlife and environmental beliefs in British Columbia. Soc Nat Resour 2(1):285–296. 10.1080/08941928909380692

[CR30] Ernstson H, Barthel S, Andersson E, Borgström ST (2010) Scale-crossing brokers and network governance of urban ecosystem services: the case of Stockholm. Ecol Soc 15(4):28

[CR31] Ernstson H, Sörlin S, Elmqvist T (2009) Social movements and eco-system services-the role of social network structure in pro-tecting and managing urban green areas in Stockholm. Ecol Soc 13(2):39

[CR32] Fischer J, Gardner T, Bennett E, Balvanera P, Biggs R, Carpenter S, Daw T, Folke C, Hill R, Hughes T, Luthe T, Maass M, Meacham M, Norströ A, Peterson G, Queiroz C, Seppelt R, Spierenburg M, Tenhunen J (2015) Advancing sustainability through mainstreaming a social-ecological systems perspective. Curr Opin Environ Sustain 2015:144–149. 10.1016/j.cosust.2015.06.002

[CR33] Freese J, Rüffer C (2005) Kooperativer Naturschutz in der Kulturlandschaft. Partizipation, Öffentlichkeitsbeteiligung, Nachhaltigkeit. In Feindt PH, Newig J (eds), Perspektiven der politischen Ökonomie (pp. 257–279), Metropolis-Verlag

[CR109] Friedkin NE (1998) A structural theory of social influence. Cambridge University Press. 10.1017/CBO9780511527524

[CR34] Friedman RS, Guerrero AM, McAllister RR, Rhodes JR, Santika T, Budiharta S, Wilson KA (2020) Beyond the community in participatory forest management: a governance network perspective. Land use policy 97: 104738. 10.1016/j.landusepol.2020.104738

[CR35] Gallemore C, Di Gregorio M, Moeliono M, Brockhaus M (2015) Transaction costs, power, and multi-level forest governance in Indonesia. Ecol Econ 114:168–179. 10.1016/j.ecolecon.2015.03.024

[CR36] Geng Y, Cao R, Han X, Tian W, Zhang G, Wang X (2022) Scientists are working overtime: when do scientists download scientific papers?. Scientometrics 127(11):6413–6429

[CR37] Girvan M, Newman ME (2002) Community structure in social and biological networks. Proc Natl Acad Sci USA 99(12):7821–7826. 10.1073/pnas.122653799 12060727 10.1073/pnas.122653799 PMC122977

[CR106] Glisson C, James LR(2002) The cross-level effects of culture and climate in human service teams. J Organ Behav 23(6):767–794

[CR38] Gorris P, Koch L (2024) Building trust in environmental co-management: social embeddedness in a contested German biodiversity conservation governance process. Environ Sci Policy 154: 103695. 10.1016/j.envsci.2024.103695

[CR39] Grendstad G, Selle P (2000) Cultural myths of human and physical nature: integrated or separated?. Risk Anal 20(1):27–40. 10.1111/0272-4332.00003 10795336 10.1111/0272-4332.00003

[CR40] Grimble R, Wellard K (1997) Stakeholder methodologies in natural resource management: a review of principles, contexts, experiences and opportunities. Agric Syst 55(2):173–193. 10.1016/S0308-521X(97)00006-1

[CR41] Guerrero AM, McAllister RRJ, Corcoran J, Wilson KA (2013) Scale mismatches, conservation planning, and the value of Social-Network Analyses. Conserv Biol 27(1):35–44. http://www.jstor.org/stable/23360331 23305381 10.1111/j.1523-1739.2012.01964.x

[CR42] Halik A, Verweij M, Schlüter A (2018) How marine protected areas are governed: a cultural theory perspective. Sustainability 10(1):252. 10.3390/su10010252

[CR43] Halik A, Verweij M, Schlüter A (2022) Deliberating coral reef protection–Cultural Theory tested. Mar Policy 139: 105036. 10.1016/j.marpol.2022.105036

[CR44] Hawcroft LJ, Milfont TL (2010) The use (and abuse) of the new environmental paradigm scale over the last 30 years: a meta-analysis. J Environ Psychol 30(2):143–158. 10.1016/j.jenvp.2009.10.003

[CR45] Herzog, LMJ (2020) Micro-pollutant regulation in the River Rhine: cooperation in a common-pool resource problem setting. Springer Nature, Cham, Switzerland

[CR47] Jasny L, Sayles J, Hamilton M, Gomez L, Jacobs D, Prell C, Matous P, Schiffer E, Guererro AM, Barnes ML (2021) Participant engagement in environmentally focused social network research. Soc Netw 66:125–138. 10.1016/j.socnet.2021.01.00

[CR48] Kaltenborn BP, Bjerke T, Strumse E (1998) Diverging attitudes towards predators: do environmental beliefs play a part?. Hum Ecol Rev 1–9. https://www.jstor.org/stable/24707187

[CR49] Knight AT, Cowling RM, Campbell BM (2006) An operational model for implementing conservation action. Conserv Biol 20(2):408–419. 10.1111/j.1523-1739.2006.00305.x 16903102 10.1111/j.1523-1739.2006.00305.x

[CR50] Krackhardt D, Stern RN (1988) Informal networks and organizational crises: an experimental simulation. Soc Psychol Q 51(2):123–140. 10.2307/2786835

[CR52] Latter B, Demski C, Capstick S (2024) Wanting to be part of change but feeling overworked and disempowered: Researchers’ perceptions of climate action in UK universities. PLOS Clim 3(1):e0000322

[CR53] Luyet V, Schlaepfer R, Parlange MB, Buttler A (2012) A framework to implement stakeholder participation in environmental projects. J Environ Manag 111:213–219. 10.1016/j.jenvman.2012.06.026 10.1016/j.jenvman.2012.06.026 22926750

[CR54] Mbaru EK, Barnes ML (2017) Key players in conservation diffusion: using social network analysis to identify critical injection points. Biol Conserv 210:222–232. 10.1016/j.biocon.2017.03.031

[CR55] Melderegister Stadt Osnabrück (2023) Bevölkerungsdaten zu verschiedenen Jahrgängen für den internen Gebrauch. Statistikstelle, Osnabrück

[CR108] Meuser M, Nagel U (2009) The expert interview and changes in knowledge production. In Bogner A, Littig B, Menz W (eds), Interviewing Experts (pp. 17–42). Palgrave Macmillan.

[CR56] Morgans CL, Guerrero AM, Ancrenaz M, Meijaard E, Wilson KA (2017) Not more, but strategic collaboration needed to conserve Borneo’s orangutan. Glob Ecol Conserv 11:236–246. 10.1016/j.gecco.2017.07.004

[CR58] Neutens T, Schwanen T, Witlox F, De Maeyer P (2010) Equity of urban service delivery: a comparison of different accessibility measures. Environ Plan a 42(7):1613–1635. 10.1068/a4230

[CR59] Newman ME, Girvan M (2004) Finding and evaluating community structure in networks. Phys Rev E 69(2):026113. 10.1103/PhysRevE.69.026113 10.1103/PhysRevE.69.026113 14995526

[CR60] Niedersächsisches Landesamt für Statistik (2021) Meine Gemeinde, meine Stadt—ausgewählte Daten auf Verwaltungseinheitsebene (VE). Retrieved from https://www.statistik.niedersachsen.de/startseite/datenangebote/meine_gemeinde_meine_stadt/meine-gemeinde-meine-stadt-uebersichtskarte-niedersachsen-100776.html

[CR61] Niemiec RM, Gruby R, Quartuch M, Cavaliere CT, Teel TL, Crooks K, Manfredo M (2021) Integrating social science into conservation planning. Biol Conserv 262: 109298. 10.1016/j.biocon.2021.109298

[CR62] Nita A, Clocanea CM, Manolache S, Rozylowicz L (2018) A network approach for understanding opportunities and barriers to effective public participation in the management of protected areas. Soc Netw Anal Min 8(1):1–11

[CR63] Noss RF (1991) Landscape connectivity: different functions at different scales. In Hudson WE (ed), Landscape linkages and biodiversity (pp 27–39). Island Press.

[CR64] O’Brien P, Prehn R, Rind N, Lin I, Choong PF, Bessarab D, Bunzli S (2022) Laying the foundations of community engagement in Aboriginal health research: establishing a community reference group and terms of reference in a novel research field. Res Involv Engagem 8(1):40. 10.1186/s40900-022-00365-7 35927687 10.1186/s40900-022-00365-7 PMC9354439

[CR65] Ogunbode CA (2013) The NEP scale: measuring ecological attitudes/worldviews in an African context. Environ Dev Sustain 15:1477–1494. 10.1007/s10668-013-9446-0

[CR66] OpenAI (2024) ChatGPT (4.0) [Large language model]. https://chat.openai.com

[CR67] Pienaar EF, Lew DK, Wallmo K (2015) The importance of survey content: testing for the context dependency of the new ecological paradigm scale. Soc Sci Res 51:338–349. 10.1016/j.ssresearch.2014.09.005 25769871 10.1016/j.ssresearch.2014.09.005

[CR68] Prell C, Hubacek K, Reed M (2009) Stakeholder analysis and social network analysis in natural resource management. Soc Nat Resour 22(6):501–518. 10.1080/08941920802199202

[CR69] Przesdzink F, Herzog LM, Fiebelkorn F (2021) Combining stakeholder-and social network-analysis to improve regional nature conservation: a case study from Osnabrück, Germany. Environ Manag 69(2):271–287. 10.1007/s00267-021-01564-w 10.1007/s00267-021-01564-w PMC8789692 34850249

[CR70] Przesdzink F, Sperling N, Oswald T, Fiebelkorn F (2024) Psychological characteristics of environmental stakeholders and interactions in their social network. Discov Sustain 5(1):177. 10.1007/s43621-024-00360-w

[CR71] Przesdzink F, Deden M, Graw J, Fiebelkorn F (2024) Analysis of the needs of various stakeholder groups with regard to efficient and low-conflict environmental protection—a case study in the Osnabrück region. Naturschutz in Praxis und Forschung, 01/2024, 40-44.10.23766/NiPF.202401.07

[CR72] Quinn RE, Rohrbaugh J (1983) A spatial model of effectiveness criteria: towards a competing values approach to organizational analysis. Manag Sci 29(3):363–377. 10.1287/mnsc.29.3.363

[CR73] Ray B, Bhattacharya R (2013) Stakeholder attitudes and conservation of natural resources: exploring alternative approaches. In Banerjee S, Chakrabarti A (eds), Development and sustainability: India in a global perspective (pp. 463–495). 10.1007/978-81-322-1124-2_20

[CR74] Reed M, Graves AR, Dandy N, Posthumus H, Klaus H, Morris J, Prell C, Quinn C, Stringer L (2009) Who’s in and why? A typology of stakeholder analysis methods for natural resource management. J Environ Manag 90:1933–1949. 10.1016/j.jenvman.2009.01.001 10.1016/j.jenvman.2009.01.001 19231064

[CR75] Richardson K, Steffen W, Lucht W, Bendtsen J, Cornell SE, Donges JF, Rockström J (2023) Earth beyond six of nine planetary boundaries. Sci Adv 9(37):eadh2458. 10.1126/sciadv.adh2458 37703365 10.1126/sciadv.adh2458 PMC10499318

[CR76] Riley LM, Kaneakua JP(2024) (Re) Building trust with indigenous communities: reflections from cultural brokers. J High Educ Outreach and Engagem 28(3):169–184

[CR77] Sanborn T, Jung J (2021) Intersecting social science and conservation. Front Mar Sci 8: 676394. 10.3389/fmars.2021.676394

[CR78] Schein EH (2010) Organizational culture and leadership, 4th edn. Jossey-Bass, San Francisco

[CR79] Schliep R, Stoll-Kleemann S (2010) Assessing governance of biosphere reserves in Central Europe. Land Use Policy 27(3):917–927. 10.1016/j.landusepol.2009.12.005

[CR80] Schuett MA, Selin SW, Carr DS (2001) Making it work: keys to successful collaboration in Natural Resource Management. Environ Manag 27(4):587–593. 10.1007/s002670010172 10.1007/s002670010172 11289456

[CR81] Schultz L, Duit A, Folke C (2011) Participation, adaptive co-management, and management performance in the world network of biosphere reserves. World Dev 39(4):662–671. 10.1016/j.worlddev.2010.09.014

[CR82] Schwarz, M, Thompson, M, 1990. Divided we stand: redefining politics, technology, and social choice. University of Pennsylvania Press

[CR83] Sinthumule NI, Ratshivhadelo T, Nelwamondo T (2020) Stakeholder perspectives on land-use conflicts in the South African section of the Greater Mapungubwe Transfrontier Conservation Area. J land use Sci 15(1):11–24. 10.1080/1747423X.2020.1739767

[CR84] Smircich L (1983) Methods of culture and organizational analysis. In: Jiménez AC (ed) The Anthropology of organizations. Routledge, pp 255–274

[CR85] Steinhäußer R, Siebert R, Steinführer A, Hellmich M (2015) National and regional land-use conflicts in Germany from the perspective of stakeholders. Land Use Policy 49:183–194. 10.1016/j.landusepol.2015.08.009

[CR86] Sulich A, Sołoducho-Pelc L, Ferasso M (2021) Management styles and decision-making: pro-ecological strategy approach. Sustainability 13(4):1604. 10.3390/su13041604

[CR87] Tambosi LR, Martensen AC, Ribeiro MC, Metzger JP (2014) A framework to optimize biodiversity restoration efforts based on habitat amount and landscape connectivity. Restor Ecol 22(2):169–177. 10.1111/rec.12049

[CR88] Torabi N, Cooke B, Bekessy SA (2016) The role of social networks and trusted peers in promoting biodiverse carbon plantings. Aust Geogr 47(2):139–156. 10.1080/00049182.2016.1154535

[CR89] UNESCO (1996) Biosphere reserves: the seville strategy and the statutory framework of the world network. UNESCO

[CR90] van Asselt MBA, Rotmans J, Den Elzen MGJ, Hilderink HBM (1995) Uncertainty in integrated assessment modelling. a cultural perspective based approach. RIVM Rapport 461502009, GLOBO report series 9

[CR92] Van Cuong C, Dart P, Hockings M (2017) Biosphere reserves: attributes for success. J Environ Manag 188:9–17. 10.1016/j.jenvman.2016.11.069 10.1016/j.jenvman.2016.11.069 27918925

[CR93] van Eijnatten FM, van der Ark LA, Holloway SS (2015) Ipsative measurement and the analysis of organizational values: an alternative approach for data analysis. Qual Quant 49:559–579. 10.1007/s11135-014-0009-8

[CR94] Vance-Borland K, Holley J (2011) Conservation stakeholder network mapping, analysis, and weaving. Conserv Lett 4(4):278–288. 10.1111/j.1755-263X.2011.00176.x

[CR95] Varvasovszky Z, Brugha R (2000) A stakeholder analysis. Health Policy Plan 15(3):338–345. 10.1093/heapol/15.3.338 11012410 10.1093/heapol/15.3.338

[CR96] Vogler D, Macey S, Sigouin A (2017) Stakeholder analysis in environmental and conservation planning. Lessons Conserv 7(7):5–16

[CR97] Von Ruschkowski E, Mayer M (2011) From conflict to partnership? Interactions between protected areas, local communities and operators of tourism enterprises in two German national park regions. J Tour Leis Stud 17(2):147–181

[CR98] Van der Wal M, De Kraker J, Offermans A, Kroeze C, Kirschner PA, Van Ittersum M (2014) Measuring social learning in participatory approaches to natural resource management. Environ policy Gov 24(1):1–15. 10.1002/eet.1627

[CR99] Wasserman S, Faust K (1994) Social network analysis: methods and applications, 8th edn. Cambridge University Press

[CR100] Weber F, Weber F (2014) Naturparke als Regionalmanager: instrumente einer grenzüberwindenden und” nachhaltigen” Regionalentwicklung?!. Nimm’s sportlich-Planung als Hindernislauf. Verlag der ARL-Akademie für Raumforschung und Landesplanung, pp 48–61

[CR101] Wielinga E, Robijn S (2020) Energising networks: tools for co-creation. BRILL

[CR102] Young J, Watt A, Nowicki P, Alard D, Clitherow J, Henle K, Richards C (2005) Towards sustainable land use: identifying and managing the conflicts between human activities and biodiversity conservation in Europe. Biodivers Conserv 14:1641–1661. 10.1007/s10531-004-0536-z

[CR105] Young JC, Searle K, Butler A, Simmons P, Watt AD, Jordan A (2016) The role of trust in the resolution of conservation conflicts. Biol Conserv 195:196–202

[CR103] Yuxi Z, Linsheng Z, Ling-en W, Hu Y (2022) Measuring the conflict tendency between tourism development and ecological protection in protected areas: a study on National Nature Reserves in China. Appl Geogr 142: 102690. 10.1016/j.apgeog.2022.102690

[CR104] Zubair L (2001) Challenges for environmental impact assessment in Sri Lanka. Environ Impact Assess Rev 21(5):469–478. 10.1016/S0195-9255(01)00081-6

