# Supplementary material for: The “Netweave-Approach”—A Platform Combining Sociology, Resource Management and Psychology for Networking Conservation Stakeholders
Source: Environ Manage. 2025 Aug 30;75(12):3283–302. doi: 10.1007/s00267-025-02268-1 (PMC12575591; doi:10.1007/s00267-025-02268-1)
Supplement: Supplementary file 1 — Consent Form for Data Collection and Use [file 267_2025_2268_MOESM1_ESM.docx]

**Consent Form for Data Collection and Use**

**Data Controller**
The data controller responsible for processing your data is the "Netweave" project at the University of Osnabrück, Department of Biology Education, reachable at: fprzesdzink@uos.de, +4915202011879, Barbarastraße 11, 49076 Osnabrück.

**Purposes of Data Processing**
The information you provide will be used to:

1. Create an organizational profile of your institution.
2. Connect your institution with other organizations in the region and promote collaboration.

**Legal Basis**
The data processing is based on your consent in accordance with Article 6(1)(a) GDPR.

**Data Retention Period**
Your data will be stored as long as necessary to fulfill the purposes mentioned above, provided there are no legal retention obligations to the contrary.

**Data Sharing**
Your data will only be shared with:

- Staff members of the "Netweave" project at the University of Osnabrück,
- Staff members of the Ecological Station Osnabrücker Land,
- The Volunteer Coordination Office at the District of Osnabrück.
  Data will only be shared with third parties with your explicit consent or when it is essential for networking purposes.

**Your Rights**
You have the right to:

- Access the stored data (Art. 15 GDPR),
- Rectify incorrect data (Art. 16 GDPR),
- Erase your data (Art. 17 GDPR),
- Restrict the processing of your data (Art. 18 GDPR),
- Object to the processing of your data (Art. 21 GDPR),
- Data portability (Art. 20 GDPR),
- Withdraw your consent at any time (Art. 7(3) GDPR).

You can exercise your rights by contacting us at any time.

**Right to Lodge a Complaint**
You have the right to file a complaint with the competent data protection supervisory authority.

**Security Measures**
Your data is protected from unauthorized access through technical and organizational measures.

By giving your consent, you agree that your data will be collected, stored, and used in accordance with the provisions outlined above.

[ ] I consent.
