# Supplementary material for: The “Netweave-Approach”—A Platform Combining Sociology, Resource Management and Psychology for Networking Conservation Stakeholders
Source: Environ Manage. 2025 Aug 30;75(12):3283–302. doi: 10.1007/s00267-025-02268-1 (PMC12575591; doi:10.1007/s00267-025-02268-1)
Supplement: Supplementary file 2 — Implementation Analysis [file 267_2025_2268_MOESM2_ESM.docx]

**Implementation Analysis**

In this section, we focus on an analysis of the implementation of the Netweave approach in our pilot region, based on the Implementation Outcome Variables by Peters et al. (2013), and provide first advice for the implementation of the Netweave Approach in other use cases.

Acceptability

Our experience shows that the initial perceived acceptability of the Netweave Approach among stakeholders was low. Approximately one-quarter of stakeholders initially declined interviews or did not respond to inquiries. During the interviews, a common sentiment was that stakeholders believed they were already well-networked and lacked the time or interest to engage with additional stakeholders. However, this negative perception often shifted when concrete resource offers were presented. Providing tangible value to the effort of contacting other stakeholders significantly increased interest, particularly during the network consultation phase. A potential issue in this context was the position of the University of Osnabrück, where the Netweave project was based, within the regional network. The university, rarely involved in environmental projects with other regional actors, is not strongly associated with environmental initiatives and may therefore lack credibility as a network facilitator. Additionally, much of the networking effort in the Netweave project was conducted by students writing their theses. These individuals, often unfamiliar to stakeholders, may not have been perceived as competent.

Stakeholders were generally unskeptical about data privacy, likely due to the assurance that their data would not be accessible to other stakeholders. Many expressed that they would not have consented to public or stakeholder-wide access to sensitive data, such as statements about other stakeholders or acknowledgments of organizational weaknesses. The intermediary role of a networking consultant, which guarantees a higher level of discretion, was therefore an essential component of the approach design. This level of trust was apparently afforded to our research group at the university and the district administration, which will subsequently manage the platform.

For future applications of the Netweave Approach, it is strongly recommended that the network consultancy be embedded within an organization that is regionally recognized and regarded as competent, trustworthy, and impartial. When contacting stakeholders, an initial brief and informative cover letter should clearly state that the goal of the network consultation is to support stakeholders in their work and provide tangible benefits. This message should be reinforced by presenting a concise example use case.

Adoption

After the conclusion of the research project, the Netweave platform will be transferred to the administration of the city and district of Osnabrück, where designated network consultants will maintain and utilize it. This transition ensures its practical adoption. Based on the findings of this project, implementing a Netweave platform in other use cases can proceed directly without requiring preliminary studies, making the process faster, more cost-effective, and more efficient. For more Details on adoption in other use cases see “Transferability”.

Appropriateness

Previous studies have shown that the organizational culture of most stakeholder organizations in the sample region tends to reflect conservative views (Przesdzink et al., 2024-2). According to earlier interviews, stakeholders are more likely to collaborate with individuals they already know and have had positive experiences with (Przesdzink et al., 2024-1). Additionally, the average age of volunteers in German nature conservation is relatively high. A study from Saxony found that the vast majority of volunteers were over 45 years old, with nearly half being over 65 (Walz et al., 2015). Similarly, the mean age of stakeholder organization representatives on the Netweave Platform is 48. Given this demographic, it is likely that a completely new, primarily digital networking approach may encounter resistance. Wagner et al. (2023) raised similar concerns regarding older conservationists' engagement with digital tools. Public authorities and associations may also resist innovation, as they often rely on rigid, standardized procedures, viewing new methods as a potential waste of time and resources (Przesdzink et al., 2024-2).

As a result, the Netweave Approach does not rely solely on a fully digital solution, contrary to the original project plan. Initially, stakeholder organizations were expected to access the platform directly and actively network with others. However, preliminary interviews revealed widespread hesitation among stakeholders, citing limited time for additional networking and insufficient IT skills to navigate a digital platform. To address this, the role of the network consultant, accessible via phone and email, was introduced later in the project. This adjustment effectively "hides" the innovation of the online platform behind the familiar and conventional format of a personal contact person for stakeholders.

Feasability

As previously mentioned, the University of Osnabrück does not hold a prominent status in environmental conservation within the district and city of Osnabrück. This posed challenges for a networking project aimed at conservation stakeholders in the region. The conservative attitudes of organizations and associations involved in nature conservation further exacerbated recruitment difficulties during data acquisition. As a result, the stakeholder database remains incomplete, complicating the identification of optimal collaboration partners in the region. For future use cases, it is strongly recommended that a Netweave platform be hosted within an organization already recognized as responsible for stakeholder networking in the respective region.

Furthermore, students as network consultants are not only perceived by stakeholders as not fitting for this task. Despite prior training, they generally lack experience in networking. Moreover, the short duration of student theses led to frequent changes in contact persons, hindering the establishment of long-term trust with stakeholders. Additionally, having different individuals enter and retrieve data from the database created inconsistencies, making it challenging to quickly and accurately interpret specific stakeholder resources and requirements.

To address these challenges, it is recommended that one or a small number of permanently employed individuals manage and maintain a Netweave platform. This approach would enable the development of trust with stakeholders and provide a deeper personal understanding of the stakeholders through direct interviews. Such personal familiarity allows for a more accurate assessment of stakeholder needs and compatibility, independent of the online system. The importance of this qualitative and intuitive "feel" for stakeholders was emphasized by all sample stakeholders involved in networking during the project.

Fidelity

The development of the stakeholder database during the research project was suboptimal from a practical standpoint, as data collection spanned over three years. During this time, data collection methods were being developed and refined concurrently, resulting in some stakeholders being contacted up to three times—once for each of the two questionnaires and once for a qualitative interview. Additionally, long intervals often occurred between the initial contact and the start of network consultancy. This led some stakeholders to perceive the project as unproductive or even bothersome. In some cases, stakeholders forgot about the project due to the prolonged periods without communication, causing inconvenience and complications during later interactions—especially when these were conducted by new personnel, as student turnover was frequent. In several instances, stakeholders declined to continue participation after the second or third contact, leaving incomplete profiles in the database.

This issue should not arise during the implementation of a new use case, as the data collection methods are now fully established. If new methods are to be added or existing ones modified to suit a specific use case, such changes should be finalized before the start of data collection. Data collection should be limited to a maximum of six months to ensure timely initiation of network consultancy, which provides concrete value to stakeholders. This timeline and process should be clearly communicated to stakeholders during the initial contact to manage expectations and foster engagement.

Implementation Cost

The work conducted during the project was carried out by university staff funded through scholarships and by students earning study credits, which minimized implementation costs. However, as recommended, establishing a new platform with permanent employees will incur specific personnel costs. The time required to manage a Netweave platform in practice depends heavily on the type and level of stakeholder interest. Nonetheless, at least one full-time position per 100 stakeholders should be anticipated.

There are no financial costs associated with using the online platform, as the necessary infrastructure is provided free of charge via GitHub (https://github.com/Netweave-Managed-Networking). Training in the setup of an own Netweave Platform as well as in the methodology for data collection, interpretation, and network consulting currently requires a one-day workshop. To streamline this process, video tutorials will be created, eliminating the need for in-person workshops and enabling users to setup and operate their own platform with minimal prior knowledge.

For stakeholders, the costs are minimal. Their primary responsibility is coordinating an interview via email, which typically lasts 30 to 40 minutes. This low effort is offset by a comparatively high benefit, as they receive tailored collaboration opportunities with individually selected partners.

Coverage

It is likely that not all nature conservation stakeholders in the Osnabrück region were included in the platform. Some stakeholders declined to participate in data collection, and many smaller, local stakeholders were likely missed due to the limitations of the snowball sampling method. Entire sectors, such as construction and climate protection, have not yet been integrated. However, a stakeholder platform like the one developed in this project can never truly be considered "complete." Theoretically, its resolution could extend to individual households, which are stakeholders of adjacent ecosystems at the smallest local scale. An individual cost-benefit analysis is essential to evaluate the time required to integrate specific sectors into network management. Depending on the use case's scope and the resources available for network management, decisions must be made regarding whether the inclusion of a particular sector justifies the effort involved. Within the scope of this research project, the maximum possible coverage was achieved with approximately 200 actors listed in the database, representing the most relevant stakeholder groups for the project’s objectives.

Sustainability

The long-term maintenance of our Netweave platform at the University of Osnabrück is uncertain due to the expiration of project funding. To address this, the platform’s sustainability was ensured through early communication with the primary actors responsible for regional environmental stakeholder management—the administrations of the city and district of Osnabrück. Both administrations have staff positions for network management. These staff members will be trained in the Netweave Approach during a workshop funded as part of the project, as detailed under "Implementation Costs."

For other use cases, at least in Central Europe, it is recommended to link a Netweave platform to a regional administrative institution. Unlike voluntary or scientific organizations, in Central Europe administrative institutions are often perceived as responsible for regional stakeholder management and possess stable financial resources. If local administrative institutions do not represent these criteria, an alternative organization should be carefully selected. Doing so ensures that the platform is recognized, actively used by stakeholders, and supported over the long term.
