# Supplementary material for: The “Netweave-Approach”—A Platform Combining Sociology, Resource Management and Psychology for Networking Conservation Stakeholders
Source: Environ Manage. 2025 Aug 30;75(12):3283–302. doi: 10.1007/s00267-025-02268-1 (PMC12575591; doi:10.1007/s00267-025-02268-1)
Supplement: Supplementary file 3 — Interview Guideline [file 267_2025_2268_MOESM3_ESM.docx]

| **Card #1** | **Section:** 1 - Preparation | **Intention:** Preparation |
| --- | --- | --- |
| - **Technical Checkup**   - **In-person Interview:**     - Is a notebook or recording device ready for use?     - Is the tablet for the online questionnaires operational and connected to the internet (is the mobile data connection functioning)?   - **Digital Interview:**     - Is a notebook or recording app ready for use?     - Is the communication tool operational?       - If necessary, provide a brief introduction to the functionality of the communication tool being used.       - Test the camera and microphone functionality. - **Ensure Interview Cards are in the Correct Order** | | |

| **Card #2** | **Section:** 2 – Small Talk | **Intention:**  Acclimatizing to the Situation |
| --- | --- | --- |
| - **Welcome the Interviewee:** Begin with a friendly and professional greeting. - **Brief Small Talk – Suggested Topics:**   - Begin with informal conversation to create a relaxed atmosphere.   - Briefly introduce yourself and the purpose of the project in a conversational tone.   - Ask general, non-project-related questions (e.g., about the participant's day or recent events) to establish rapport. - **Thank the Participant:** Express gratitude for their time and for attending. | | |

| **Card #3** | **Section:** 3 – Information about the Interview | **Intention:**  Procedure and Framework Conditions |
| --- | --- | --- |
| - **Brief Self-Introduction:**   - Introduce yourself, including your name, fields of study, and mention your participation in the Netweave research project. - **Introduce the purpose of the interview:**   - Explain that the research project focuses on environmental conservation in the Osnabrück region.   - Emphasize the importance of collaboration among various environmental stakeholders.   - Mention that a database is being created to facilitate "networking consultations."   - **Provide a practical example:**     - Explain how the database connects stakeholders who have complementary knowledge or resources.   - **Specify the participant's role:**     - State that the participant has been invited as a representative to provide insights into their organization. - **Interview Duration:**   - Inform the participant about the expected duration of the interview (20–30 minutes). - **Voluntary Participation:**   - Emphasize voluntary participation:     - Inform the participant that the interview is entirely voluntary and can be stopped at any time.   - Explain the use of interview cards:     - Mention the use of interview cards to maintain structure.     - Reassure the participant that glancing at the cards is just to stay organized.     - Optionally, briefly show the cards to the participant. - **Recording Consent:**   - **Explain the purpose of recording:**     - The recording will be reviewed and transcribed.     - Only the transcriptions will be used for analysis.     - Data will remain confidential, inaccessible to third parties, and not directly shared with other stakeholders.     - Data will be used solely for networking purposes, such as connecting stakeholders with complementary resources or competences.   - Ask if the participant has any remaining questions.   - **Request explicit consent to record:**     - In-person: Hand over the consent form for signature.     - Online: Request a verbal "Yes" to record their consent. | | |

| **Card #4** | **Section:** 4 – Main Phase | **Intention:** Internal Foci |
| --- | --- | --- |
| - **Introduce the topic:**   - Remind the participant that the interview focuses on their organization (NAME) and potential collaborations with other organizations. - **Start with an open question:**   - Ask the participant to describe their organization:     - "Who is your institution, and what makes it unique?"     - Encourage them to share their focus areas (e.g., social, thematic, political), activities, roles, or goals. - **Ask about support capabilities:**   - "What competencies or resources could your organization potentially offer to support other stakeholders?" - **Ask about support requirements:**   - "Where could your organization benefit from the support of other stakeholders?" | | |

| **Card #5** | **Section:** 4 – Main Phase | **Intention:** External Foci |
| --- | --- | --- |
| - **Explore collaboration preferences:**   - "What does someone need to do to align well with your organization?"   - Encourage responses regarding social, thematic, or political aspects of collaboration. - **Identify traditional allies:**   - "Are there types of organizations you would describe as your 'traditional allies'?" - **Discuss deal-breakers:**   - "What behaviors or characteristics are absolute deal-breakers for collaboration with your organization?"   - Probe for social, thematic, or political conflict triggers. - **Identify traditional adversaries:**   - "Are there types of organizations you consider 'traditional adversaries'?" | | |

| **Card #6** | **Section:** 5 – Online-Questionnaire | **Intention:** Bearbeitung der Online Fragebögen |
| --- | --- | --- |
| - **Transition to digital questionnaire:**   - Thank the participant for their responses and introduce the digital questionnaire.   - Explain that it covers standardized aspects that don't require open-ended responses. - **Provide instructions based on the interview format:**   - **In-person:**     - "The questionnaire is on this tablet. Please navigate through it at your own pace. Let me know if you have any questions or encounter any issues." (Hand over the tablet)   - **Online:**     - "I've shared the link to the questionnaire in the chat. Please click the link to access it. Let me know if you have any questions or encounter any issues." (Share the link in the chat) - **Support during questionnaire completion:**   - Be attentive in case the participant experiences technical difficulties.   - Offer additional assistance if needed, particularly for participants who may not be tech-savvy. | | |

| **Card #7** | **Section: 6** – Wrap-Up | **Intention:** Wrap-Up |
| --- | --- | --- |
| - **Wrap-Up Question:**   - Thank the participant for their responses so far.   - Ask: "Before we finish, is there anything you’d like to add that we haven’t discussed today? Do you have any questions for us?" - **Closing the Interview:**   - Conclude by expressing gratitude: "I’d like to sincerely thank you for your time and participation. Your input has been extremely helpful, and we hope to provide you with valuable cooperation opportunities very soon." | | |
