# Supplementary material for: The “Netweave-Approach”—A Platform Combining Sociology, Resource Management and Psychology for Networking Conservation Stakeholders
Source: Environ Manage. 2025 Aug 30;75(12):3283–302. doi: 10.1007/s00267-025-02268-1 (PMC12575591; doi:10.1007/s00267-025-02268-1)
Supplement: Supplementary file 4 — List of Resource Categories [file 267_2025_2268_MOESM4_ESM.docx]

**Complete List of Resource Categories Used in the Netweave Platform for the Osnabrück Region (Translated from German)**

- **Education/Didactics**
  - **Definition:** Dissemination and transfer of knowledge about environmental conservation, environmental education, and Education for Sustainable Development (ESD).
  - **Anchor Quote:** "Rather to pass on knowledge, motivation, energy, and sometimes even creative pedagogical ideas. For example, we recently organized a spring festival with the local heritage association in Achmer. We built a small marble track and created quiz cards on two nature-related topics. We also set up ring toss games where participants threw rings at waterfowl images and then identified or guessed the bird species. Activities like these are enjoyable and can be passed on to others."
- **Agricultural Biological Knowledge**
  - **Definition:** Expertise in agricultural practices.
  - **Anchor Quote:** "With knowledge about agriculture, which is quite complex."
- **General Biological Knowledge**
  - **Definition:** Broad biological expertise, such as species conservation, biodiversity, environmental assessments, and expert opinions.
  - **Anchor Quote:** "I have good foundational knowledge in many areas."
- **Amphibians & Reptiles Biological Knowledge**
  - **Definition:** Expertise on Amphibians and Reptiles and their interactions with humans.
  - **Anchor Quote:** "Herpetology. I also conduct amphibian studies, such as using pitfall traps and funnel traps for newts and tadpoles."
- **Soil Biological Knowledge**
  - **Definition:** Expertise on the living, uppermost layer of the Earth's crust.
  - **Anchor Quote:** "Our expertise lies in soil and soil protection—everything related to soil."
- **Fish Biological Knowledge**
  - **Definition:** Expertise on fish and their interactions with humans.
  - **Anchor Quote:** "We have people trained as professional fishers."
- **Aquatic Ecosystems Biological Knowledge**
  - **Definition:** Expertise related to still or flowing water ecosystems. Keywords: lakes, rivers.
  - **Anchor Quote:** "Combating harmful impacts on aquatic ecosystems, including supporting measures to preserve the landscape and natural watercourses."
- **Grassland/Meadows Biological Knowledge**
  - **Definition:** Expertise on pastures and fields with grasses or herbaceous plants. Keywords: flowering meadows, orchard meadows.
  - **Anchor Quote:** "The lake is surrounded by over 3,000 hectares of extensively used grasslands, including wet meadows actively restored with the conservation administration over the past 25-30 years."
- **Microorganisms Biological Knowledge**
  - **Definition:** Expertise on microscopic organisms invisible to the naked eye. Examples: bacteria, viruses.
  - **Anchor Quote:** "We conduct microbial analyses, including soil bacteria assessments, which help determine soil health and its suitability for agriculture."
- **Peatland Biological Knowledge**
  - **Definition:** Expertise on areas with peat-forming vegetation and wet habitats. Keywords: raised bogs, fens.
  - **Anchor Quote:** "Our expertise includes extensive knowledge about ecosystems in the region, from flowing waters and lakes to fen and raised bog landscapes."
- **Plant Biological Knowledge**
  - **Definition:** Expertise on plants and their interactions with humans.
  - **Anchor Quote:** "Regular activities like orchard pruning."
- **Mammal Biological Knowledge**
  - **Definition:** Expertise on mammals and their interactions with humans.
  - **Anchor Quote:** "[...] everything creeping and crawling, including mammals like bats, hares, and deer."
- **Urban Ecosystems Biological Knowledge**
  - **Definition:** Expertise on ecosystems in urban areas with significant human influence.
  - **Anchor Quote:** "Our work focuses on creating green corridors in cities to connect isolated urban habitats and improve biodiversity in heavily anthropogenic environments."
- **Bird Biological Knowledge**
  - **Definition:** Expertise on birds and their interactions with humans.
  - **Anchor Quote:** "We have people with expertise in ornithology [...] and more."
- **Forest Biological Knowledge**
  - **Definition:** Expertise on deciduous and mixed forests, forestry practices.
  - **Anchor Quote:** "It's about implementing good forestry practices with local foresters."
- **Invertebrates Biological Knowledge**
  - **Definition:** Expertise on multicellular animals without a spine. Examples: bees, snails, jellyfish.
  - **Anchor Quote:** "We conducted a crayfish stocking initiative with the NDR a few years ago, introducing noble crayfish to a lake where they have thrived."
- **Bureaucratic/Legal Competencies**
  - **Definition:** Administrative, legal, financial, and management skills.
  - **Anchor Quote:** "In our local group, we have a lawyer who can handle legal topics effectively, even though environmental law isn’t his specialty."
- **Influence**
  - **Definition:** Political or decision-making influence, especially at the local level.
  - **Anchor Quote:** "We can influence zoning plans, requiring certain green spaces or rooftop greenery in collaboration with municipalities."
- **Financial Resources**
  - **Definition:** Funding and financial support.
  - **Anchor Quote:** "We now provide financial resources for projects like establishing flowering meadows."
- **Land**
  - **Definition:** Agricultural and outdoor spaces.
  - **Anchor Quote:** "We essentially provide land as our main resource."
- **Human Resources**
  - **Definition:** Employees, volunteers, youth, and members.
  - **Anchor Quote:** "We have a large team ready to assist with any project that needs hands-on support."
- **Contacts**
  - **Definition:** Specific contact details and networks.
  - **Anchor Quote:** "We have a large distribution network, reaching 14,000 students and 2,000 staff members."
- **Machinery/Equipment**
  - **Definition:** Includes farm machinery, lab equipment, and drones.
  - **Anchor Quote:** "We’ve acquired forestry machinery over decades, enabling small forest owners to manage their forests."
- **Public Relations**
  - **Definition:** Public outreach, marketing, and visibility.
  - **Anchor Quote:** "We are often seen as representing public interests and are asked for statements on environmental topics."
- **Facilities**
  - **Definition:** Buildings, rooms, and laboratories.
  - **Anchor Quote:** "As a university, we can offer rooms like lecture halls, even if not permanent office space."
- **Other Competencies**
  - **Definition:** Specialized knowledge outside biology (e.g., geology) and project management experience.
  - **Anchor Quote:** "We combine theoretical knowledge with practical planning and implementation experience."
- **Mediation**
  - **Definition:** Conflict resolution and stakeholder connection services.
  - **Anchor Quote:** "We mediate between environmental protection stakeholders and leaseholders to protect nesting birds during breeding seasons."
- **Additional Resources**
  - **Definition:** Miscellaneous resources like grant approvals, structural funding, and land management.
