# Supplementary material for: The “Netweave-Approach”—A Platform Combining Sociology, Resource Management and Psychology for Networking Conservation Stakeholders
Source: Environ Manage. 2025 Aug 30;75(12):3283–302. doi: 10.1007/s00267-025-02268-1 (PMC12575591; doi:10.1007/s00267-025-02268-1)
Supplement: Supplementary file 5 — Online Questionnaire (OCAI, NEP, MoN) [file 267_2025_2268_MOESM5_ESM.docx]

**Questionnaire (Translated from German)**

**General Information about Your Organization**

- Does your organization have a defined spatial or thematic jurisdiction?
- How many members (for associations) or employees (for authorities, companies, or scientific groups) does your organization have? (Please provide an estimate)

**Organizational Culture (Sliding Scale: 0–100)**

In addition to content-based aspects (e.g., shared interests, complementary resources), social factors also influence how collaborations function. We aim to understand where the focus of participating organizations lies—for example, stability versus flexibility, adherence to formalities versus rapid goal achievement—and how they "harmonize" or complement one another in collaborations. The following questions relate to the "organizational culture" of your organization—its working methods, success criteria, and internal focus.

1. **Dominant Characteristics of Your Organization**
   Assign a score from 0 (does not apply at all) to 100 (applies very strongly) for each statement:
   - The organization is a very personal place, like an extended family. People seem to share a lot about themselves.
   - The organization is very dynamic and entrepreneurial. People are willing to take risks and stand up for their ideas.
   - The organization is highly results-oriented. A primary concern is getting the job done. People are competitive and performance-driven.
   - The organization is very controlled and structured. Formal procedures generally dictate what people do.
2. **Organizational Leadership**
   Assign a score from 0 (does not apply at all) to 100 (applies very strongly) for each statement:
   - Leadership is generally characterized by mentoring, support, or encouragement.
   - Leadership is generally characterized by entrepreneurship, innovation, or risk-taking.
   - Leadership is generally characterized by a pragmatic, aggressive, and results-oriented focus.
   - Leadership is generally characterized by coordination, organization, or smooth operational efficiency.
3. **Management of Employees**
   Assign a score from 0 (does not apply at all) to 100 (applies very strongly) for each statement:
   - The management style is characterized by teamwork, agreement, and participation.
   - The management style is characterized by individual risk-taking, innovation, freedom, and uniqueness.
   - The management style is characterized by fierce competitiveness, high demands, and performance pressure.
   - The management style is characterized by job security, conformity, predictability, and stability in relationships.
4. **Organizational “Glue”**
   Assign a score from 0 (does not apply at all) to 100 (applies very strongly) for each statement:
   - The glue that holds the organization together is loyalty and mutual trust. Commitment to the organization is strong.
   - The glue that holds the organization together is a focus on innovation and development. Staying ahead of trends is valued.
   - The glue that holds the organization together is an emphasis on achievement and goal attainment.
   - The glue that holds the organization together is formal rules and policies. Maintaining smooth operations is important.
5. **Strategic Focus of Your Organization**
   Assign a score from 0 (does not apply at all) to 100 (applies very strongly) for each statement:
   - The organization places great importance on developing its people. There is a high level of trust, openness, and participation.
   - The organization emphasizes acquiring new resources and creating new challenges. Experimentation and exploring new opportunities are encouraged.
   - The organization prioritizes competitive actions and achievements. Meeting targets is a primary focus.
   - The organization values sustainability and stability. Efficiency, control, and smooth operations are critical.
6. **Success Criteria of Your Organization**
   Assign a score from 0 (does not apply at all) to 100 (applies very strongly) for each statement:
   - The organization defines success based on human resource development, teamwork, employee engagement, and care for individuals.
   - The organization defines success through unique or innovative accomplishments. It is a product leader and innovator.
   - The organization defines success based on profitability and outperforming competitors. Market leadership is key.
   - The organization defines success based on efficiency. Reliable delivery, smooth scheduling, and cost-effective production are crucial.

**Environmental Worldviews (5-Point Likert Scale)**

The following statements reflect general attitudes toward the relationship between humans and nature. Please indicate the extent to which your organization agrees with each statement. These questions address opinions—there are no "right" or "wrong" answers. Different environmental worldviews exist among stakeholder groups and can impact how collaborations function. We assess the alignment and potential conflicts in environmental attitudes to recommend suitable partnerships.

- Humanity is approaching the limit of the Earth’s capacity to support its population.
- Humans have the right to modify the natural environment to suit their needs.
- When humans interfere with nature, the consequences are often catastrophic.
- Human ingenuity will ensure that the Earth remains livable.
- Humanity is exploiting the natural environment.
- The Earth has enough resources if we learn how to increase them.
- Animals and plants have the same right to exist as humans.
- Nature’s balance is strong enough to cope with the impacts of modern industrial societies.
- Despite our unique abilities, humans are subject to the laws of nature.
- The so-called "ecological crisis" facing humanity is greatly exaggerated.
- The Earth is like a spaceship with limited space and resources.
- Humans are destined to rule over the rest of nature.
- The balance of nature is delicate and easily upset.
- Someday, humans will learn enough about nature to control it.
- Without significant changes, a major ecological disaster is inevitable.

**Myths of Nature**

Below, you will find four simplified representations of the balance of nature. Each diagram depicts nature as a line and a ball balancing on it. These representations illustrate different abilities of nature to withstand disturbances.


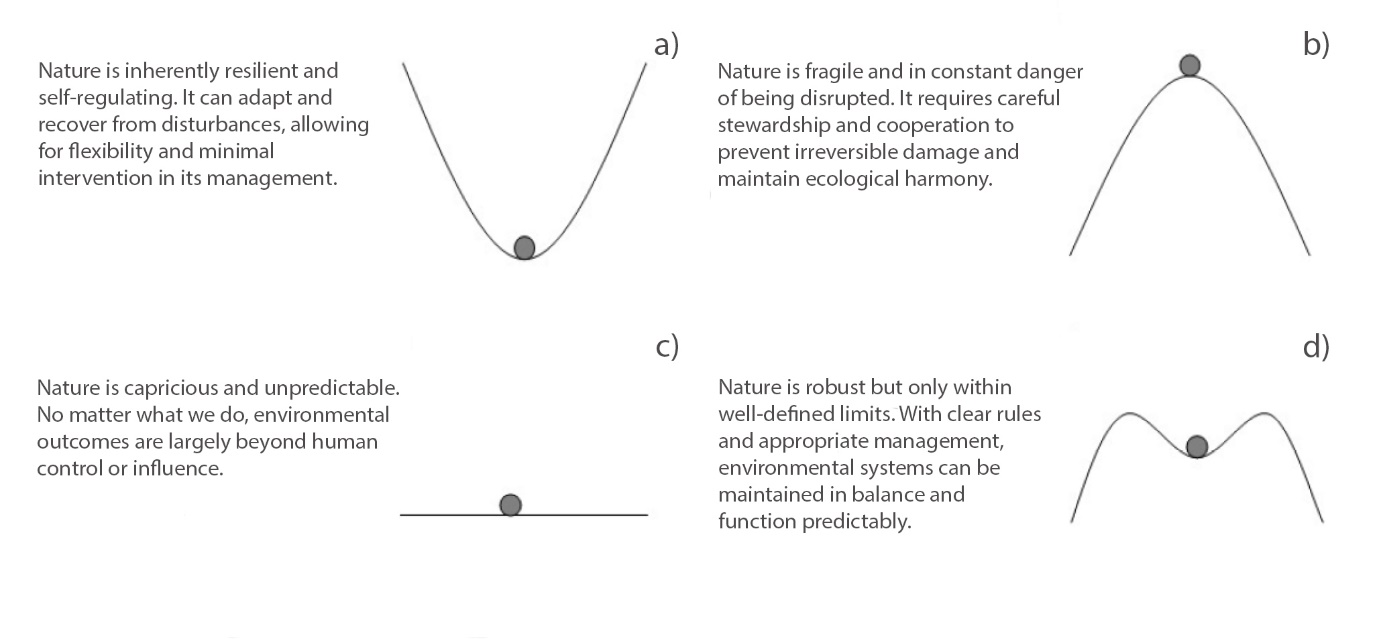


**Feedback and Comments**

Do you have any feedback or comments about this questionnaire?
