# Supplementary material for: The “Netweave-Approach”—A Platform Combining Sociology, Resource Management and Psychology for Networking Conservation Stakeholders
Source: Environ Manage. 2025 Aug 30;75(12):3283–302. doi: 10.1007/s00267-025-02268-1 (PMC12575591; doi:10.1007/s00267-025-02268-1)
Supplement: Supplementary file 6 — Stakeholder Platform Frontend [file 267_2025_2268_MOESM6_ESM.docx]

**Stakeholder Platform – Frontend**

The landing page, shown in Fig. 1, provides access to participation links for two online questionnaires: the Social Network Analysis and the psychological aspects (OCAI, NEP, MoN; see Supplementary Material “Online Questionnaire (OCAI, NEP, Mon)”). This page is used on our tablets during data collection interviews or sent to stakeholders for digital data collection. Screenshots in Fig. 1 and subsequent figures are from a platform with a dummy database featuring fictional stakeholders. This ensures the privacy of our stakeholders and allows for an English-language representation. The actual platform used in the Osnabrück region operates in German.

***
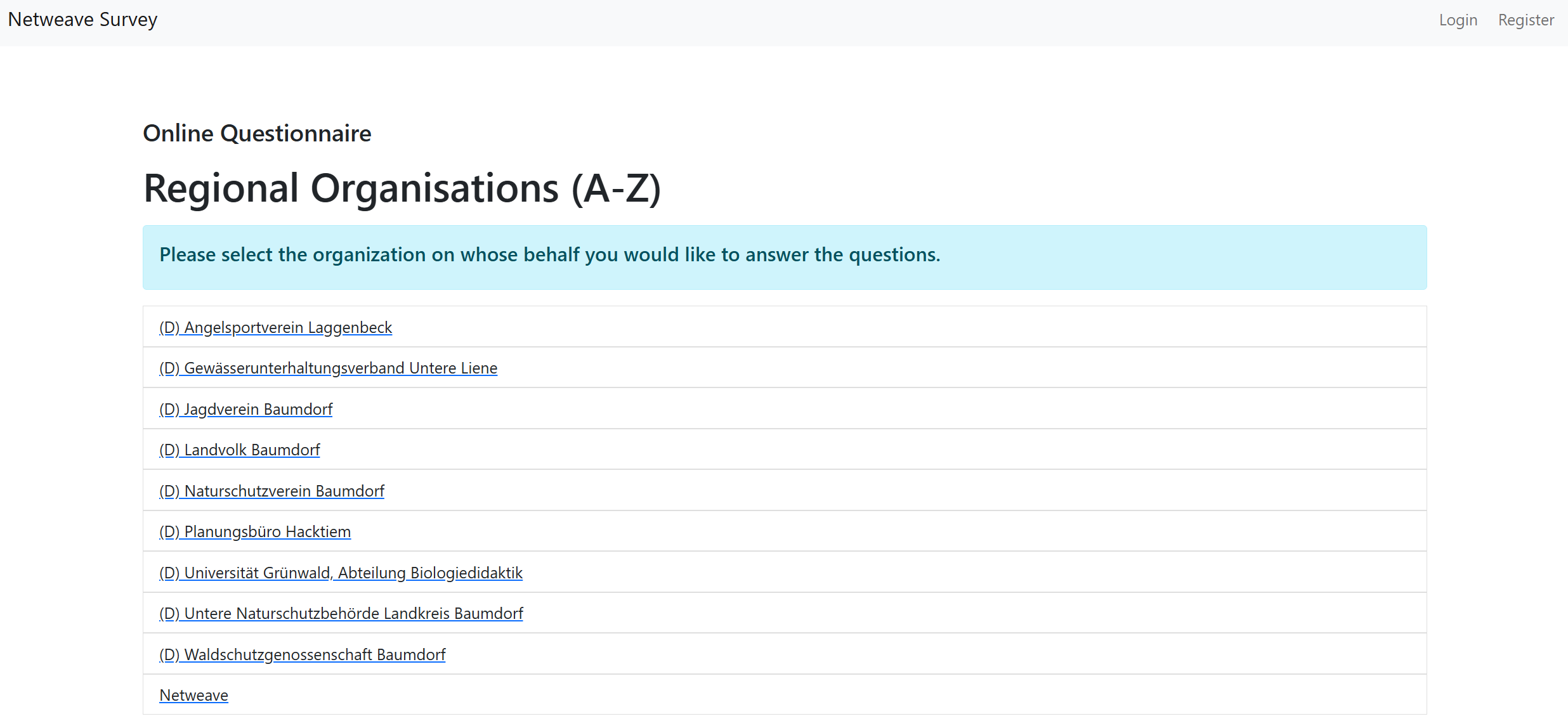
***

**Figure 1: Landing Page of a Netweave Platform.** Network consultants can register or log in using the buttons in the top right corner. Stakeholder organizations select their organization and proceed to complete the provided online questionnaires.

Upon logging in, users access the main section of the platform, as shown in Figure 2, which is exclusively available to network consultants.


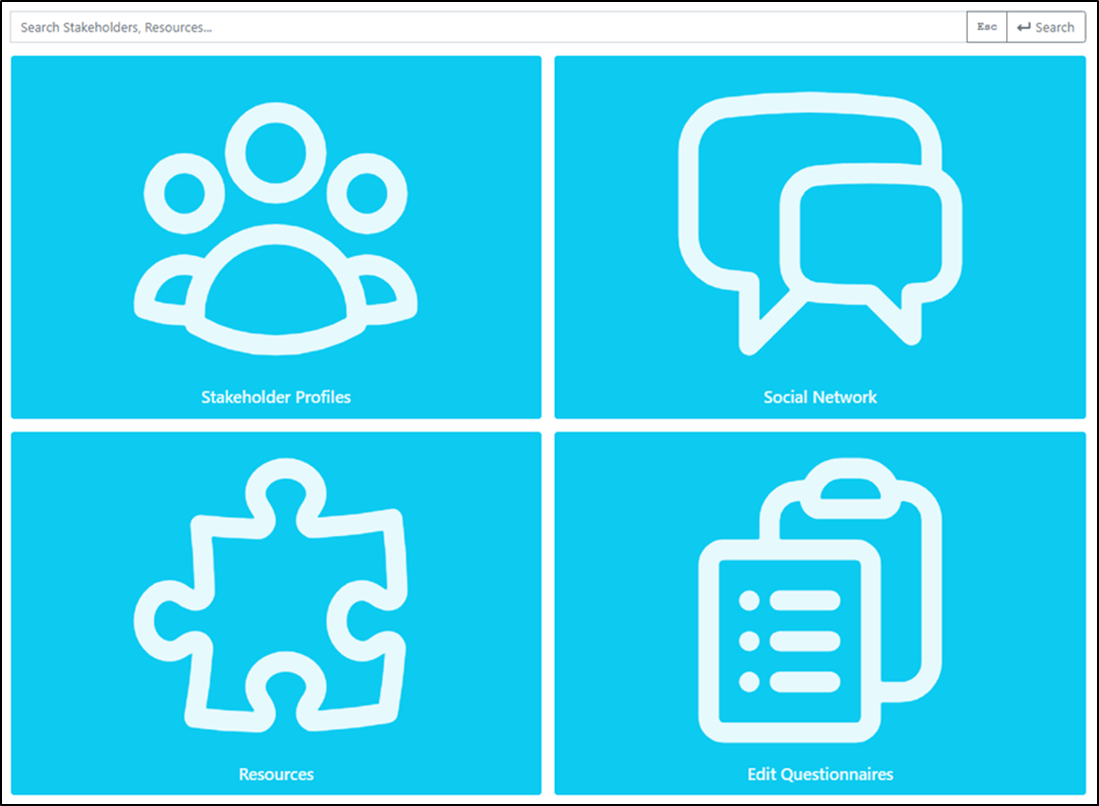


**Figure 2: Main Menu of a Netweave Platform.** The search bar at the top allows users to query the entire database, for example, to locate specific resources (see Fig. 4). In the blue section, the button in the top left provides access to an overview of all stakeholders listed in the database, with links to their profiles (see Fig. 3a&b). The button in the top right enables the download of cross-tables showing stakeholder interactions across the dimensions of acquaintance, collaboration, and conflict. These tables can be imported into network analysis programs like Gephi or UCINET for in-depth analyses or visualization of network graphs. The bottom left section allows users to view and edit all resource categories in the database, while the bottom right section facilitates editing of the deployed online questionnaires.

A core feature of the main section is the stakeholder profiles, which consolidate all data collected from stakeholders. Profiles include contact information, social network data, resources (and resource requirements), Organizational Culture, NEP scores, MoN preferences, spatial and thematic limitations, criteria for or against collaborations, and typical allies and adversaries. At the bottom of each profile, stakeholders who can meet the resource requirements of the profile owner are automatically suggested. An example stakeholder profile is shown in Figures 3a and 3b.


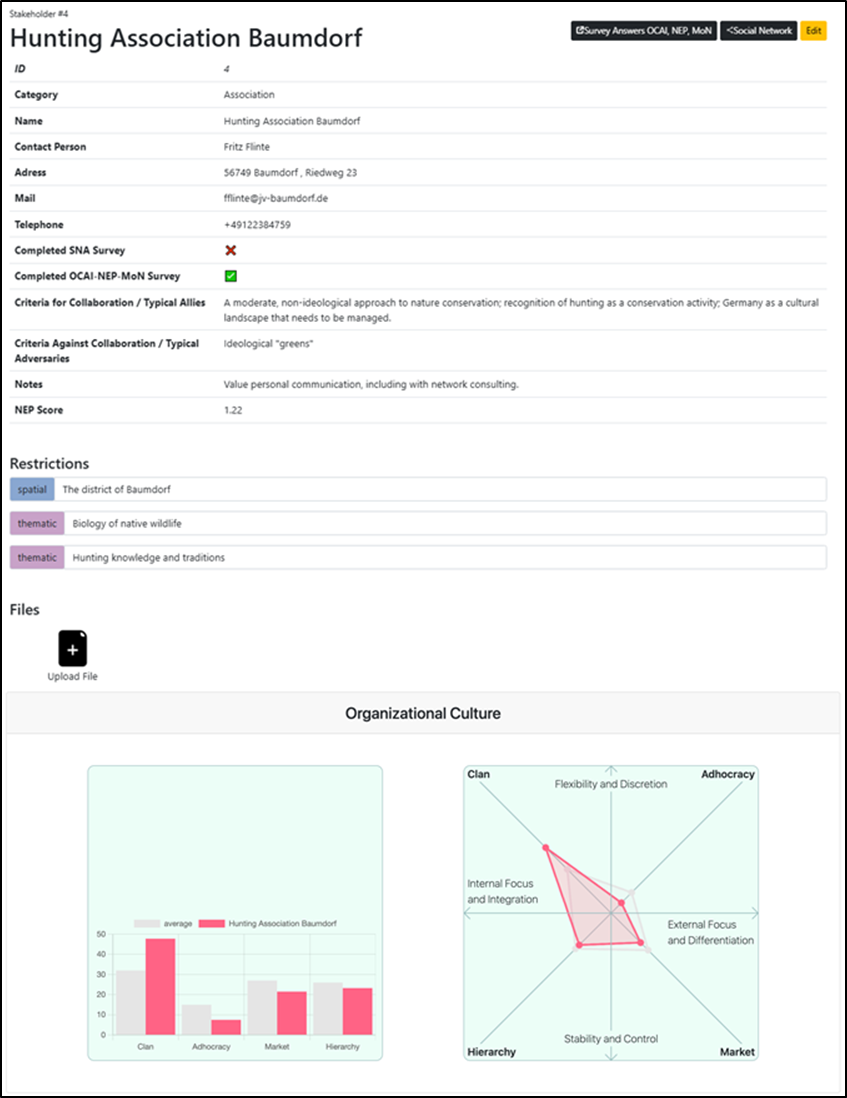


**Figure 3a: Top section of a stakeholder profile.** The black buttons in the top right provide access to this stakeholder’s responses to the OCAI, NEP, and MoN questionnaires, as well as their interactions with other stakeholders—their social network. The yellow “Edit” Button leads to a page where all contents of the stakeholder profile can be manually edited (see section “Stakeholder Edit Page”). The “Upload” button allows users to attach files, such as organizational charts or interview transcripts. Below this section, a radar chart displays the values of the four organizational cultures within the stakeholder organization (in pink) compared to the average values of all organizations in the database (in gray).


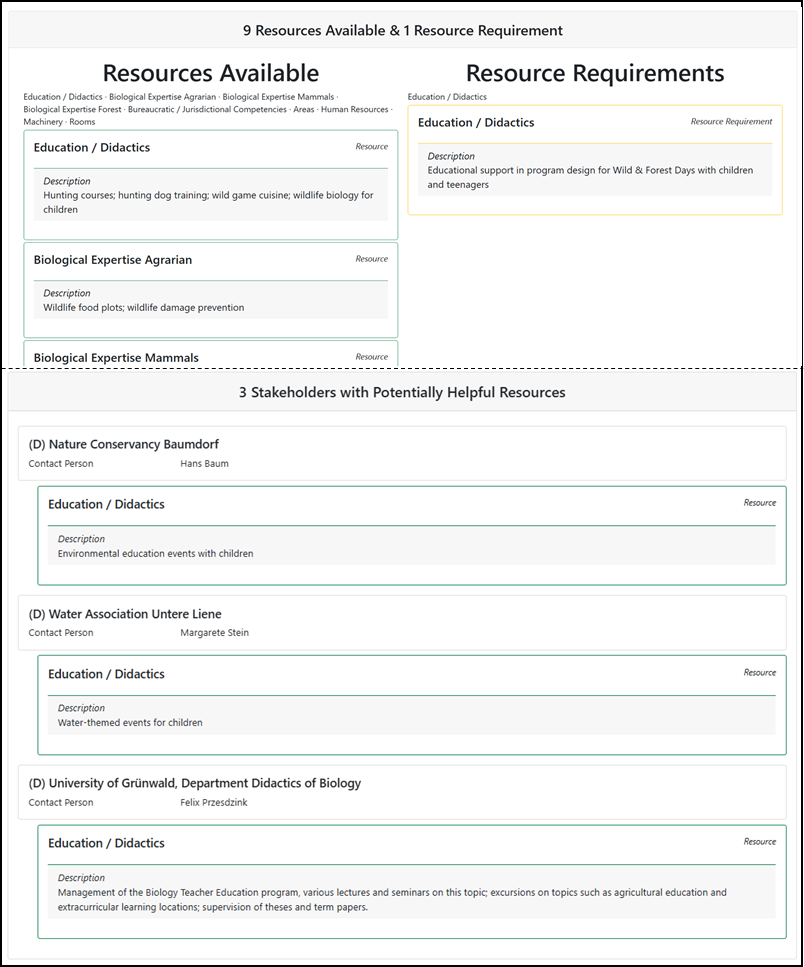


**Figure 3b: Bottom Section of a Stakeholder Profile.** Below the section shown in Fig. 3a, the stakeholder’s available resources and resource requirements are displayed (upper part of this figure). The presentation of the nine total resources has been limited in the screenshot to two, with a third partially visible below. Further down, stakeholders are suggested whose resources could meet the requirements of this stakeholder.

Another essential feature of the main section is the search function (see Fig. 4). This free-text search allows users to query the entire database. Results include stakeholders whose names contain the search term, stakeholders with available resources matching the search term in their description, and resource categories with descriptions containing the search term.

**
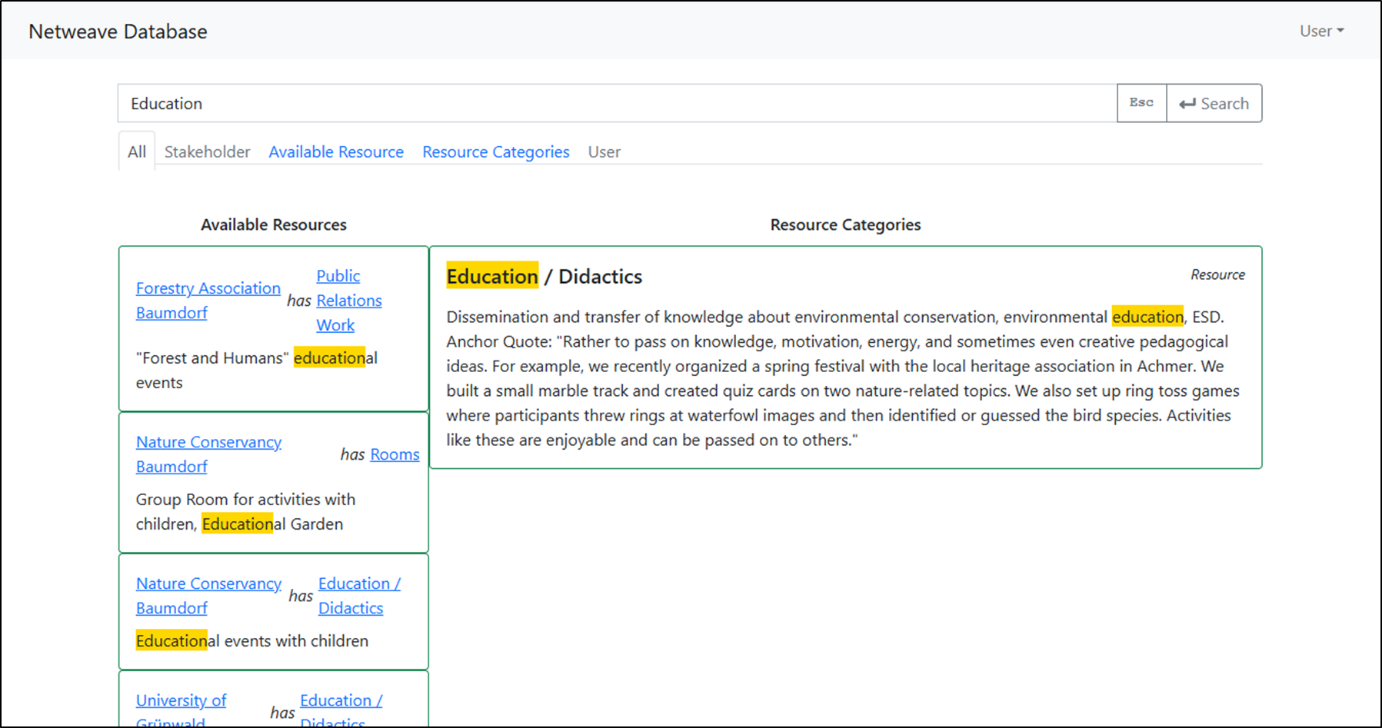
**

**Fig.4: Search results for "Education" in a Netweave platform.** The search bar at the top displays the entered term, along with an "Escape" button to clear the search and a "Search" button to execute it. Results include stakeholders (bottom left) with resources that match the term in their description, limited here to three, with a fourth truncated. Resource categories containing the term are shown on the bottom right. In this example, no stakeholders have the term in their name; if present, they would be displayed further down on the left. Clicking on stakeholders or resources takes users to their respective detail pages.


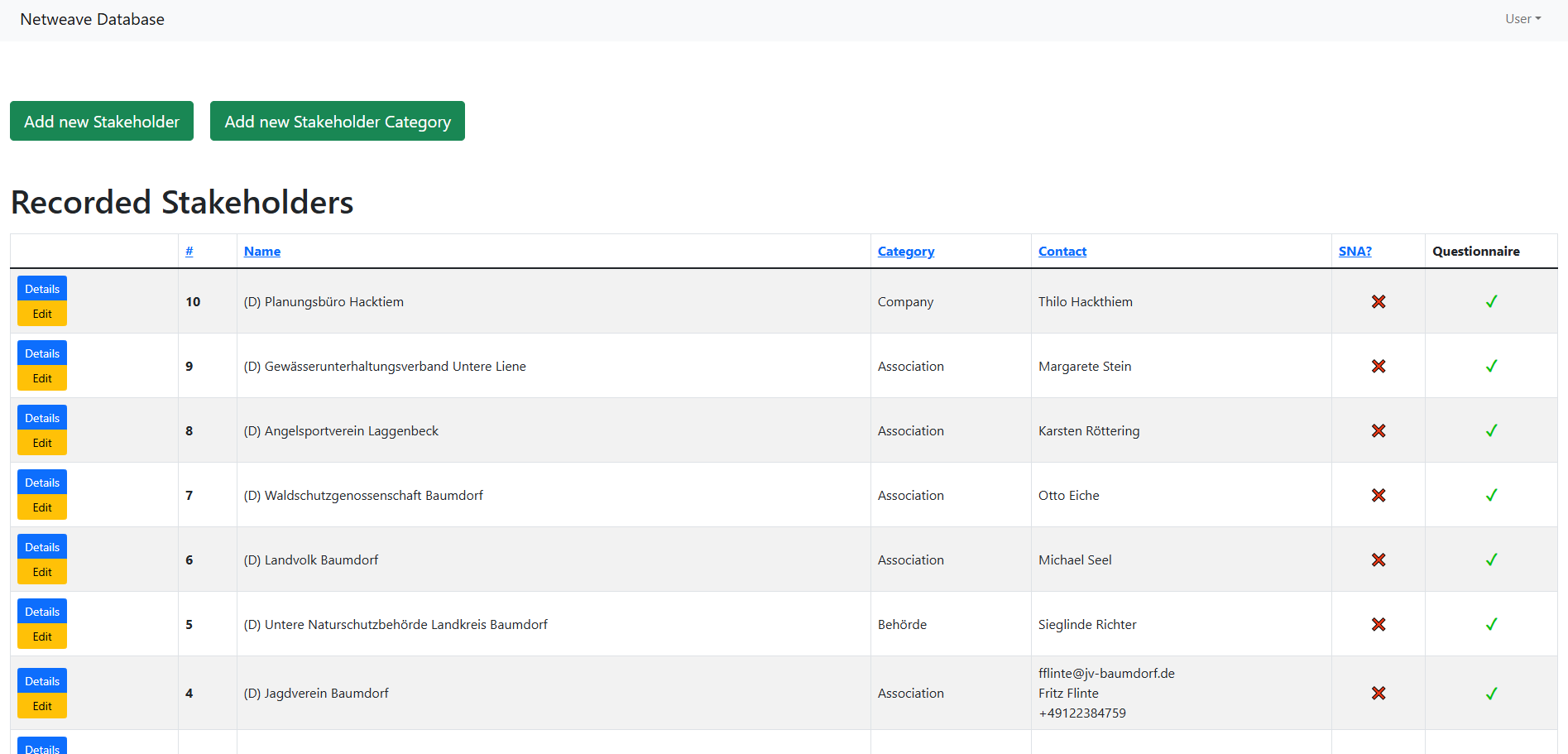


**Stakeholder Edit Page**


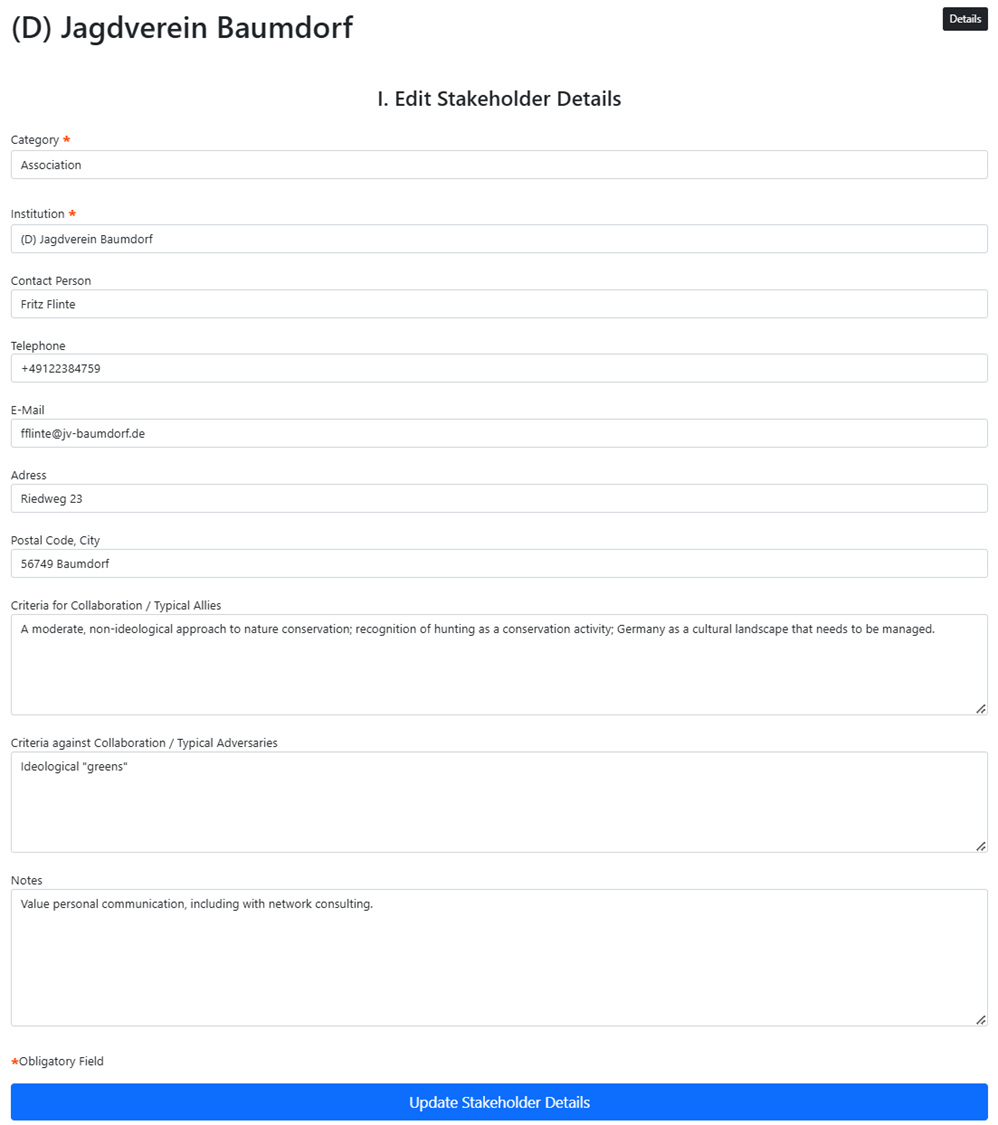


**Fig. 6: First section of the Stakeholder Edit Page.** All text fields can be freely edited. Clicking "Update Stakeholder Details" saves the changes.


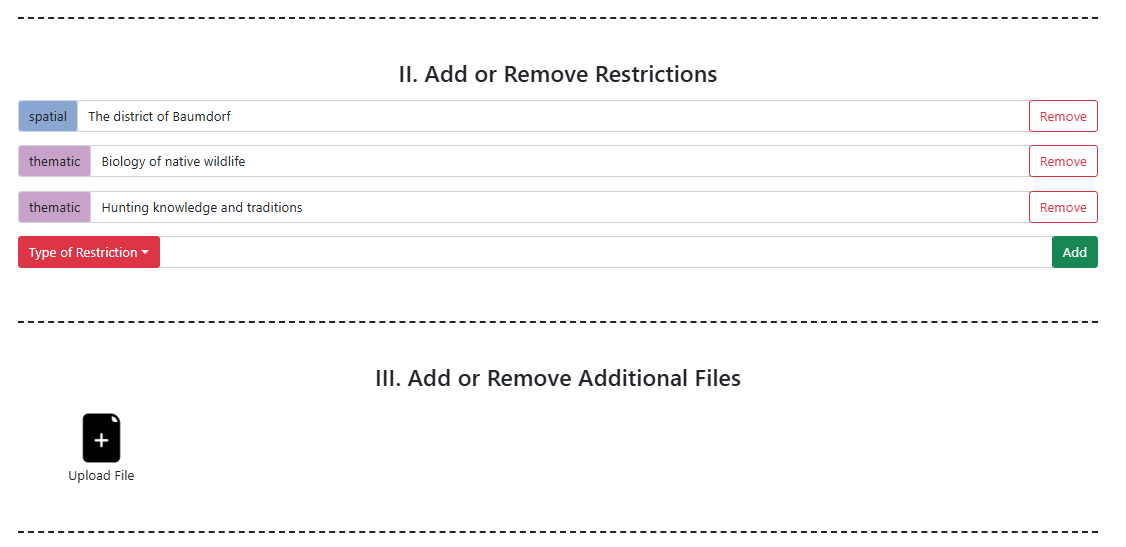


**Fig. 7: Second and third sections of the Stakeholder Edit Page.** In the second section, spatial and thematic limitations can be added, described, and removed. In the third section, additional files, such as organizational charts as image files, Word or PDF transcripts, or audio recordings of interviews, can be uploaded.


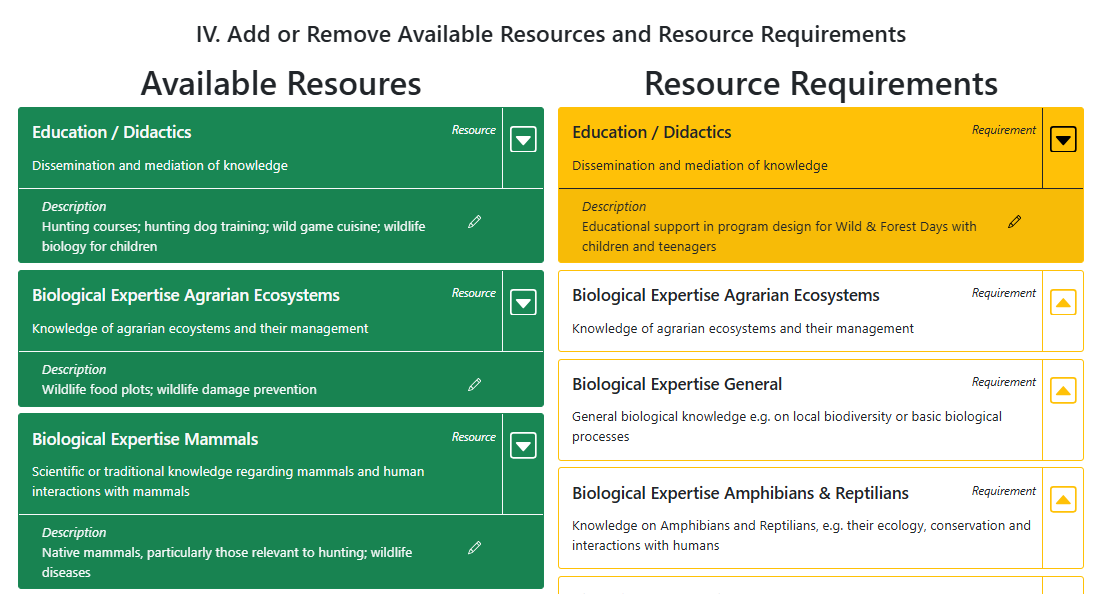


**Fig. 8: Fourth section of the Stakeholder Edit Page.** Colored elements indicate resources already assigned to this stakeholder, which can be removed by clicking the arrow in the top-right corner of the element. The description of each resource can be edited by clicking the pencil icon. Uncolored elements are not assigned to the stakeholder but can be added by clicking the arrow in the top-right corner of the element. The pop-up window that appears after clicking is shown in the next image. Below, the Available Resources and Resource Requirements areas each display a list of all resource categories recorded in the platform.


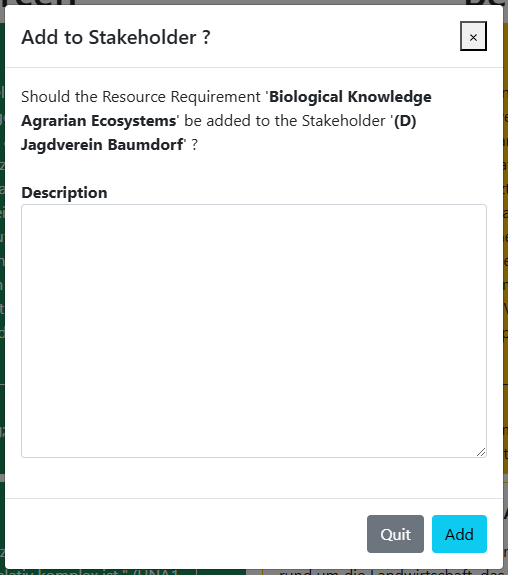


**Fig. 9:** **Adding Available Resources or Resource Requirements**. This pop-up window appears when adding an available resource or a resource requirement. A detailed description can be added in the text field.
